# Module organization and variance in protein-protein interaction networks

**DOI:** 10.1038/srep09386

**Published:** 2015-03-23

**Authors:** Chun-Yu Lin, Tsai-Ling Lee, Yi-Yuan Chiu, Yi-Wei Lin, Yu-Shu Lo, Chih-Ta Lin, Jinn-Moon Yang

**Affiliations:** 1 Institute of Bioinformatics and Systems Biology, National Chiao Tung University, Hsinchu, Taiwan; 2 Department of Biological Science and Technology, National Chiao Tung University, Hsinchu, Taiwan

## Abstract

A module is a group of closely related proteins that act in concert to perform specific biological functions through protein–protein interactions (PPIs) that occur in time and space. However, the underlying module organization and variance remain unclear. In this study, we collected module templates to infer respective module families, including 58,041 homologous modules in 1,678 species, and PPI families using searches of complete genomic database. We then derived PPI evolution scores and interface evolution scores to describe the module elements, including core and ring components. Functions of core components were highly correlated with those of essential genes. In comparison with ring components, core proteins/PPIs were conserved across multiple species. Subsequently, protein/module variance of PPI networks confirmed that core components form dynamic network hubs and play key roles in various biological functions. Based on the analyses of gene essentiality, module variance, and gene co-expression, we summarize the observations of module organization and variance as follows: 1) a module consists of core and ring components; 2) core components perform major biological functions and collaborate with ring components to execute certain functions in some cases; 3) core components are more conserved and essential during organizational changes in different biological states or conditions.

The assembly of protein complexes in time and space is essential for performing biological processes, such as cell cycle control and transcription^1^. The protein assembly can be regarded as a module, which often governs specific processes and is autonomous in relation to other parts of the organism^2^,^3^. Many works have been proposed to study the biological properties and modularity of the module. These works employed experimental methods^1^,^4^, network topology^5^,^6^, gene expression-based methods^2^,^7^, and evolutionary-based methods^8^. In addition, the modules can be approximately divided into functional module^3^, variational module^3^, and evolutionary module^9^,^10^. A functional module is a group of proteins that semi-autonomously assemble together to perform discrete physiological functions. Moreover, the proteins and protein-protein interactions (PPIs) in a module often change over seconds to assemble and disassemble for performing biological functions, as well as evolve over millions of years as proteins and PPIs are gained and lost^11^. Investigations of underlying module organization and variance are urgently required for understanding the cellular processes and module evolution.

As complete genomes become increasingly available, systems biology approaches based on homologous PPIs and modules across multiple species provide an opportunity to explore organization, evolution, and variance of modules. For investigating the modularity of the yeast cell machinery, an experimental genome-wide screen approach, based on the isoforms of complexes, was proposed and 491 complexes were identified^1^. These complexes differentially combined with attachment proteins to execute time–space potential functions in yeast. In addition, functionally interacting proteins have been shown to be gained or lost together during genome evolution^12^. However, functional modules showed limited conservation during evolution^9^. The causes of restricted evolutionary modularity need to be clarified. Previously, we inferred the module family, which consists of a group of homologous modules, from complete genomic database (e.g. Integr8) through PPI families^13^,^14^. Based on the module families and PPI families, we have reconstructed module-module interaction networks (called MoNetFamily^15^) in vertebrates. However, the understanding of module organization and variance in PPI networks is incomplete.

To address these issues, we propose PPI evolution score (PPIES) and interface evolution score (IES) as the basis to study the module organization and variance in PPI networks using module families and PPI families across multiple species. We utilized PPIES and IES to identify core and ring components of a module. Furthermore, we define protein functional variance (PFV) and module organizational variance (MOV) of PPI networks to measure the functional diversities of proteins and modules, respectively. For a module, the core proteins and PPIs are often conserved and consistently play the essential role for performing biological functions. Conversely, ring proteins and PPIs are not often conserved in module families. Compared with ring proteins, core proteins are essential for survival and preferentially constitute hubs of a PPI network. Moreover, core PPIs were co-expressed significantly more than ring PPIs in 7,208 *Homo sapiens* gene expression sets from Gene Expression Omnibus (GEO)^16^. Finally, we applied genome-wide investigations to describe the link from PFV and MOV values to module variance and biological functions in time and space. We believe that our results are useful for understanding the module organization and variance in PPI networks.

## Results and Discussion

### Overview

**Figure 1:**
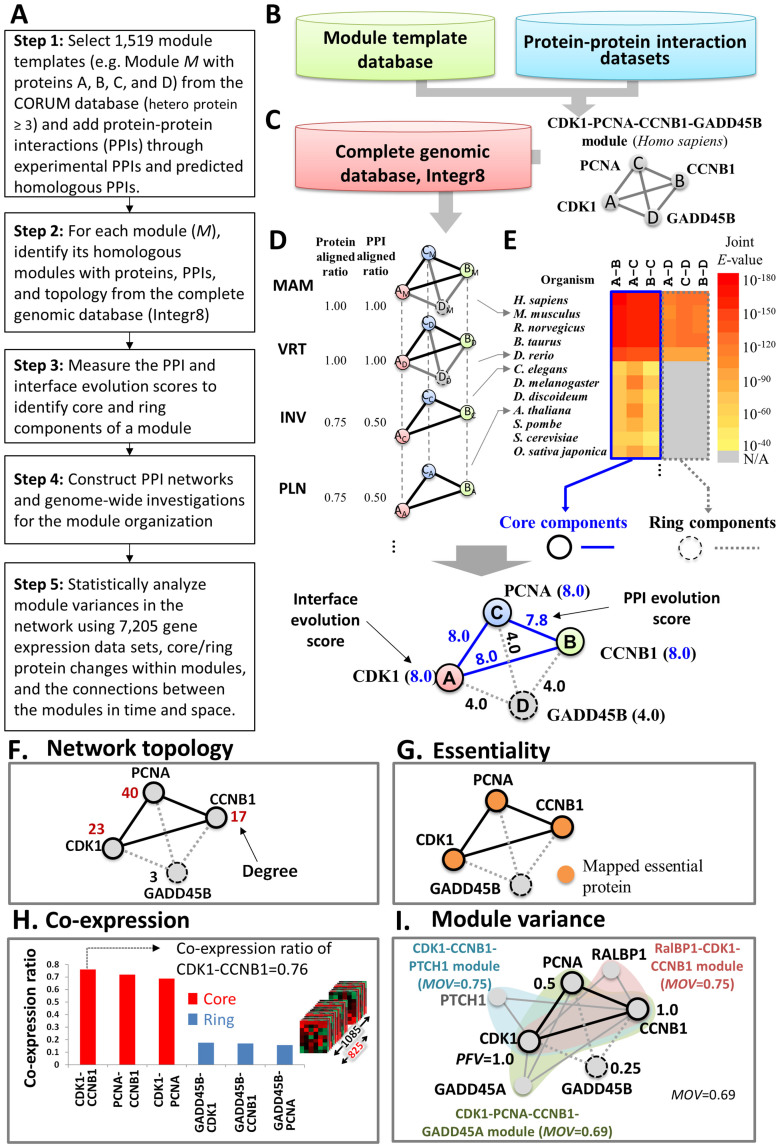
Overview of core and ring components of modules using the human CDK1–PCNA–CCNB1–GADD45B module as a template. (A) The main procedure. (B) Module template database and protein–protein interaction (PPI) database for inferring homologous PPIs. (C) Homologous PPIs and proteins of the template by searching the complete genomic database (Integr8). (D) Homologous modules of the CDK1–PCNA–CCNB1–GADD45B module. (E) PPI profiles and core (solid circle and line) and ring (dash circle and line) components of this module family across multiple organisms commonly used in molecular research projects. (F) The degrees of core and ring proteins of the module in the human PPI network, including 2,391 proteins and 11,181 PPIs. (G) Essentiality of core (solid circle) and ring (dash circle) proteins in this module family; orange circles indicate mapped essential proteins when they are homologs of essential proteins. (H) Co-expressions of six PPIs (Pearson's *r* ≥ 0.5) of this module were statistically derived from 7,208 microarray data sets. (I) The supermodule comprised four modules, including CDK1–PCNA–CCNB1–GADD45B, CDK1–CCNB1–PTCH1 (blue), CDK1–PCNA–CCNB1–GADD45A (green), and RalBP1–CDK1–CCNB1 (red), with their module organizational variance (MOV). The protein functional variance (PFV) of core proteins (e.g. CDK1 = 1.0) of this module are higher than those of ring proteins (i.e. GADD45B = 0.25).

Figure 1 shows the details of our method for identifying core and ring components of modules, and for elucidating module organization through template-based homologous modules (module families) using the following steps (Fig. 1A): First, a module template database comprising 1,519 protein complexes was selected from the Comprehensive Resource of Mammalian protein complexes database (CORUM; release 2.0)^4^. Internal PPIs of module templates were then added to templates that lacked PPIs using template-based homologous PPIs, including experimental PPIs from IntAct^17^, BioGRID^18^, DIP^19^, MIPS^20^, and MINT^21^, and predicted homologous PPIs^14^,^22^ (Fig. 1B). For each PPI of a module, we inferred its PPI family with joint *E*-values of ≤10^−40^ 14 by searching a complete genomic database (Integr8 version 103, containing 6,352,363 protein sequences in 2,274 species^23^) using previously identified homologous PPIs^14^,^22^ (Fig. 1C). Subsequently, we utilized MoNetFamily^15^ to identify homologous modules of module templates according to topological similarities across multiple species (Fig. 1D and Supplementary Text S1). Module profiles were then constructed for module families, and protein and PPI components were computed (Fig. 1E). Next, we then derived PPIES and IES scores to extrapolate core and ring components of a module. Finally, we constructed PPI networks and genome-wide investigations for organization of a module, including network topology (Fig. 1F), gene essentiality (Fig. 1G), gene expression profiles (Fig. 1H), and module variance (Fig. 1I).

### Core and ring components of a module

Homologous modules (a module family) provide the clues to understand the evolution and conserved functions of proteins and PPIs in a module. Thus, we proposed PPIES and IES to identify core and ring components of a module by utilizing homologous PPIs and proteins^15^. To derive homologous modules across multiple species, we collected 1,519 high-quality module templates (≥3 proteins in a template), which are manually annotated protein complexes from the MIPS CORUM database^4^. These 1,519 modules are selected from *H. sapiens* (1,094), *M. musculus* (248), *R. norvegicus* (148), and *B. Taurus* (29), respectively. Based on these module templates and the thresholds of functional and topology similarities^15^ (Supplementary Fig. S1 and Text S1), we inferred 58,041 homologous modules in 1,678 species from 461,077 sequence-based PPI families and 86,252 structure-based PPI families^13^,^14^. Furthermore, we reconstructed the human PPI network by these 1,515 human modules, including 1,094 human CORUM modules and 421 human homologous modules derived from the other species.

To identify core/ring proteins and PPIs of a module, we used the PPIES and IES scores to measure the protein and PPI conservations, respectively, based on 1,678 species and six taxonomic divisions (see Methods). These six divisions include mammals (MAM), vertebrates (VRT), invertebrates (INV), plants (PLN), bacteria (BCT), and archaea (ARC) according to the National Center for Biotechnology Information (NCBI) taxonomy database^24^. In a module family, a PPI with high PPIES indicates that its homologous PPIs are highly conserved across species and taxonomic divisions. In addition, IES of the protein *i* was set to the maximum PPIES of these PPIs, which reflected interactions between the protein *i* and its partners. Based on analyses of network topology, gene essentiality, and gene co-expression, we considered proteins with IES ≥ 7 and PPIs with PPIES ≥ 7 as core components of a module, and other proteins and PPIs are the ring components.

We used the CDK1–PCNA–CCNB1–GADD45B module family as an example to illustrate core and ring components and their biological properties (Figs. 1D and 1E). The core components of the CDK1–PCNA–CCNB1–GADD45B module (CORUM ID: 5545^25^) included three proteins (solid circles; i.e. cyclin-dependent kinase 1 (CDK1), proliferating cell nuclear antigen (PCNA), and G2/mitotic-specific cyclin-B1 (CCNB1)), with IESs of 8.0, and three PPIs (solid lines; i.e. CDK1–CCNB1 and CDK1–PCNA with PPIESs of 8.0, and CCNB1-PNCA with a PPIES of 7.8). Ring components (dashed circles and lines) consist of the growth arrest and DNA damage-inducible protein (GADD45) with an IES of 4.0 and three PPIs (GADD45–CDK1, GADD45–PCNA, and GADD45–CCNB1) with PPIESs of 4.0. During the G2/M cell cycle phase, GADD45B specifically interacts with the CDK1–CCNB1 complex, but not with other CDK–Cyclin complexes, to regulate activation of G2/M cell cycle checkpoint^25^.

According to six PPI profiles of CDK1–PCNA–CCNB1–GADD45B module family across several organisms that are commonly used in molecular research projects (Fig. 1E), we found that PPI families of three core PPIs (i.e. CDK1–CCNB1, CKD1–PCNA, and CCNB1–PNCA) were highly conserved. For example, the interaction between CDK1 and CCNB1 is conserved across 67 species as observed from the homologous PPIs of the human CDK1–CCNB1 PPI (confirmed by protein kinase assays^17^ and co-immunoprecipitation experiments^19^). During the G2 cell cycle phase, the active CDK1–CCNB1 interaction can enhance chromosome condensation and nuclear envelope breakdown to separate the centrosomes^26^. During the response to DNA damage, PCNA (another core protein) recruits at the replication fork to coordinate DNA replication, and activates DNA repair and damage tolerance pathways. However, no homologs of GADD45B (ring protein) were found in chloroplasts or bacteria. GADD45B is involved in G2/M cell cycle arrest, acting as an inhibitor of the CDK1–CCNB1 complex in some cases (e.g. exposure of cells to genotoxic stress)^25^.

A module is a fundamental unit formed with highly connected proteins and often possesses specific biological functions. To assess the connectivity and shared biological functions of two types (core and ring component) of module components and three module types (module template, homologous module, and the respective extended module), we computed connectivity (*C_t_*) and average relative specificity similarity (AvgRSS) scores of Gene Ontology (GO) terms (Supplementary Figs. S2 and S3; Texts S2 and S3). Among 1,519 module templates, the average *C_t_* value of core components was significantly higher than those of the others, including the ring components (Mann–Whitney *U* test, *P* = 7e-6), whole module templates (*P* = 6e-17), and extended modules (*P* = 9e-250; Supplementary Fig. S2A). Similarly, the core components of homologous modules have significantly higher average *C_t_* value than those of ring components (*P* = 1e-40), whole homologous modules (*P* = 1e-14), and extended modules (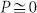; Supplementary Fig. S3A). These results indicate that the core components of the modules have high connectivity. In addition, our results also indicate that the core components often regulate similar biological processes and are localized to the same cellular compartment (Supplementary Text 3).

### Network topology of core and ring components

**Figure 2:**
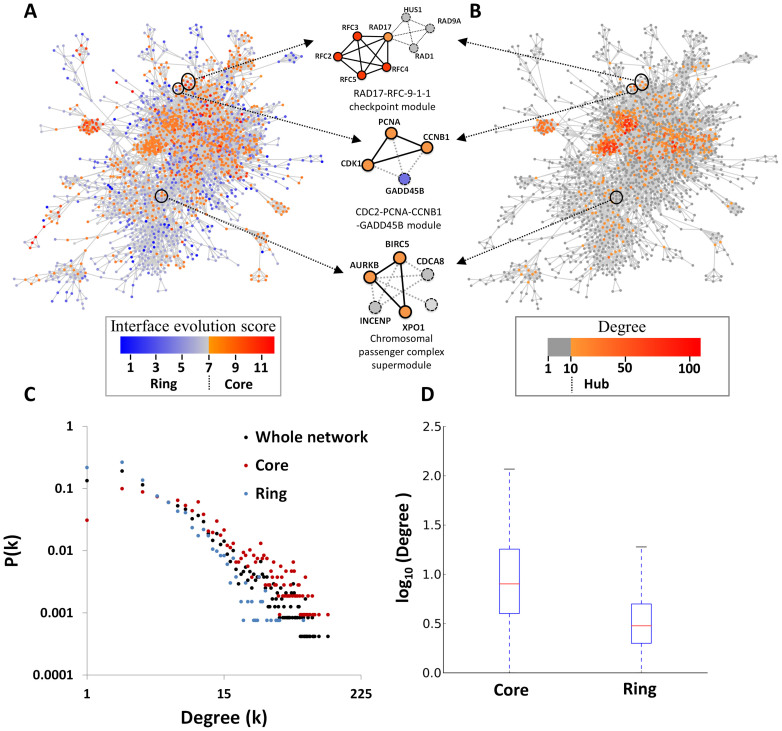
Topologies of core and ring proteins in the human protein–protein interaction network comprising 2,391 proteins and 11,181 PPIs. Each protein of the network is annotated with (A) interface evolution scores (IES) and (B) degrees. Chromosomal passenger complex supermodule, RAD17-RFC-9-1-1 checkpoint module, and CDK1-PCNA-CCNB1-GADD45B module are indicated. The core proteins, such as RFC2, RFC3, RFC4, and RFC5 of RAD17-RFC-9-1-1 checkpoint module, are often hubs (top 25% of the highest degree) of this network. Core proteins (red) have higher degrees than ring proteins (blue), and constitute the hubs of PPI networks. (C) Node degree distributions of all proteins (black), core proteins (red), and ring proteins (blue) in this scale-free PPI network. (D) The boxplot of degrees (log_10_ ratio) between core and ring proteins.

To analyze core and ring components in PPI networks, we derived a human PPI network from 1,515 homologous modules. This PPI network comprised 2,391 proteins and 11,181 PPIs (Figs. 2A and 2B), and was evaluated based on the characteristic of scale-free networks that can be described as *P*(*k*) ~ *k^−r^*, in which the probability of a node with *k* links decreases as the node degree increases on a log–log plot (Fig. 2C). The degree exponent γ was 1.60 in this PPI network, which was consistent with the architecture of previously described cellular networks^27^,^28^. Figure 2C shows the distribution of node degrees for core proteins, ring proteins, and all proteins in this human PPI network. For 1,069 core proteins, 1,322 ring proteins, and 2,391 proteins of this PPI network, the distribution of node degrees of core proteins (median is 8) was significantly higher than that of ring proteins (median is 3; *P* = 1e-117; Fig. 2D).

On the basis of a previous study^29^, we considered proteins within the top 25% of the highest degree (here, degree ≥ 10) as hubs of the network. The IES distribution of these core proteins was consistent with the hub distribution of this PPI network, particularly at the center of the network (Figs. 2A and 2B). Moreover, 43% of core proteins with degrees of ≥10 were hubs, and only 12% of ring proteins were hubs. Interestingly, node degrees of ring proteins in modules were lower than those of all proteins in this network, indicating that core proteins but not ring proteins play major roles in high connectivity of module sub-networks. Our results suggest that core proteins are preferential constituents of network hubs, as reflected by protein IES values. This observation is consistent with a previous study showing that highly conserved enzymes in a metabolic network were frequently highly connected at the center of the network and were involved in multiple pathways^30^. In the CDK1–PCNA–CCNB1–GADD45B module, the core proteins CDK1, CCNB1, and PCNA had higher degrees (≥17) than the ring protein GADD45B (degree = 3) in the human PPI network (Figs. 1F, 2A, and 2B).

### Essentiality and composition of core/ring components

Essential genes (or proteins) are considered to be required to support cellular life and likely to be common to all cells^31^. To evaluate essentiality of core and ring proteins in module families, we collected 11,384 essential proteins over 25 species from the Database of Essential Genes (DEG; version 6.5)^32^, including 8 eukaryotes (e.g. *H. sapiens* and *S. cerevisiae*) and 17 prokaryotes (e.g. *Escherichia coli* and *Bacillus subtilis*). Because homologs of essential proteins are likely to be essential, module proteins were considered essential when they were homologous to those recorded in DEG. For example, CCNB1 is a mapped essential protein and is homologous to essential proteins B_M_ (G2/mitotic-specific cyclin-B1 in mouse) and B_D_ (cyclin B1 in zebrafish) from DEG (Figs. 1D and 1G). For the CDK1–PCNA–CCNB1–GADD45B module family, homologs of the core proteins CDK1, CCNB1, and PCNA were essential proteins according to DEG^32^. In contrast, all homologs of the ring protein GADD45B were non-essential (Fig. 1G).

**Figure 3:**
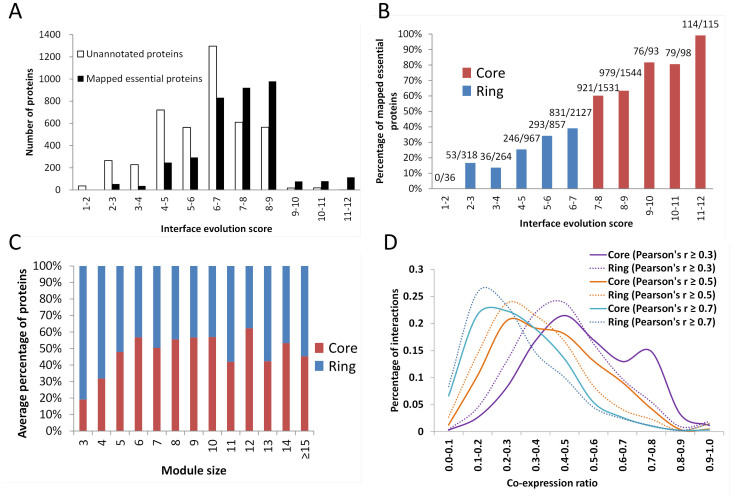
Characteristics of module organization. (A) Interface evolution score (IES) distributions of the numbers of unannotated (white) and mapped essential proteins (black). (B) The relationship between IES values and percentages of mapped essential proteins, showing rapidly increases when the IES is ≥7 (red). The proteins with IES values of ≥7 and <7 were considered core proteins (red) and ring proteins (blue), of a module, respectively. (C) The relationship between module sizes and core/ring composition of modules; percentages of core and ring components in different module sizes are similar. (D) Distributions of co-expressions of core PPIs (solid lines) and ring PPIs (dot lines) of 1,515 human modules based on Pearson's *r* thresholds of ≥0.3, ≥0.5 and ≥0.7, respectively. Co-expressions of core protein pairs are significantly higher than those of ring protein pairs.

According to the DEG data set, 7,950 proteins from 1,519 module templates were clustered into two groups, including 3,628 mapped essential proteins and 4,322 unannotated proteins without homologous protein in DEG. Among these mapped essential proteins, IES values of 60% are more than 7 and their IES values are significantly higher than those of unannotated proteins (Mann–Whitney *U* test, *P* = 3e-217; Fig. 3A). In addition, percentages of mapped essential proteins were correlated with IES (Pearson's *r* = 0.98) and these increased rapidly with IES ≥ 7 (Fig. 3B).

Based on these 11,384 essential proteins, we derived 160 essential GO molecular function (MF) terms (Supplementary Table S1, Supplementary Figs. S4 and S5, and Supplementary Text 4) and analyzed functional annotations of core and ring components using hypergeometric distributions (P ≤ 0.05). The distribution of occurrence ratios of these 160 terms between the core component set and the essential protein set is similar (Pearson's *r* = 0.77), and Pearson's *r* is 0.49 between the ring component and the essential protein set (Supplementary Fig. S5). Specifically, both core and essential protein sets have some significant MF terms, such as “structural constituent of ribosome,” “ATPase activity,” “nucleoside-triphosphatase activity,” and “chromatin binding.” These terms commonly relate to processes that are critical for survival and are conserved in the modules.

In addition, we analyzed 1,212 unannotated core proteins (IES ≥ 7; Table 1) using orthologs from the PORC database^23^ and these 160 essential GO MF terms. Among these, 462 (38%) were orthologous to essential proteins or were annotated with at least one of the 160 essential GO MF terms. Furthermore, 303 unannotated core proteins (25%) possessed child annotations of the 160 essential GO MF terms; therefore, were considered essential. Moreover, 76% and 100% of the unannotated core proteins with IES ≥ 9 or 11, respectively, were annotated with orthologs of essential proteins, were one of 160 essential GO MF terms, or were child annotations of the 160 essential GO MF terms (Table 1). These results show that protein IES provides biological insights, and that core components are often essential for survival, as indicated in DEG and GO.

Figure 3C shows the relationship between module sizes and core/ring compositions of modules. In a module, the number of core components is similar (~50%) to the number of ring components when the module size ≥5. We next analyzed the distributions of three kinds of modules: including core-only module, ring-only module, and core-ring module. Interestingly, the percentages of core-only modules were often less than 18% and were much lower than those of ring-only modules (Supplementary Fig. S6). In the previous studies, functional modules showed limited conservation during evolution, with approximately 40% of 1,161 prokaryotic modules displaying evolutionary cohesion (i.e. genes in a module tend to be gained/lost together in evolution)^9^,^10^. The present results suggest that these functional modules contain core and ring components (~50% each), and only core proteins may contribute for the evolutionary cohesion of the module. In addition, the core proteins of a module play the key role for the conservation of functional modules during evolution.

### Gene co-expression of core and ring components

Dynamic assembly and cooperation of proteins in time and space is essential for biological processes in a cell. In this study, we found that modules can be organized into core and ring components, which represent temporal and spatial conservation of dynamic PPIs and proteins. Genome-wide gene expression profiles are descriptive of molecular states that are associated with various responses to environmental perturbations and cellular phenotypes^33^. Thus, to observe the variance of PPIs and proteins in a module, we collected 7,208 *H. sapiens* gene expression data sets (≥3 samples) from GEO^16^ (Supplementary Figs. S7A and S7B). For each module among 1,519 templates, we initially selected gene expression sets that contain all proteins in this module, and evaluated co-expressions of intra-module PPIs to construct a correlation matrix (Supplementary Fig. S7C). To confirm that modules in the data sets were associated with biological functions, we selected gene expression sets that give rise to comparatively high protein expression and contain at least one co-expression of intra-module PPIs with Pearson's *r* values of ≥*h* (see Methods).

Figure 3D shows relationships between co-expression ratios (CE) with Pearson's *r* values of ≥0.3, 0.5, and 0.7 and percentages of core PPIs and ring PPIs for 1,515 human modules. When Pearson's *r* values were ≥0.3, the average CE (0.51) of interacting core proteins (core PPIs) was significantly higher than that (0.44) of interacting ring proteins (ring PPIs; Mann–Whitney *U* test, *P* = 3e-79). Similarly, when Pearson's *r* values were ≥0.7, the CE of interacting core proteins remained significantly higher than the ratio of interacting ring proteins (P = 3e-14). For example, the core PPIs CDK1–PCNA, PCNA–CCNB1, and CDK1–CCNB1 in the CDK1–PCNA–CCNB1–GADD45B module had significantly higher CEs (≥0.69) than those of the ring PPIs (≤0.18) CDK1–GADD45B, CCNB1–GADD45B, and PCNA–GADD45B, according to 1,085 high expression profile sets for this module (Fig. 1H). These results indicate that core PPIs of modules are co-expressed more frequently than ring PPIs, suggesting that core components are often simultaneously active or inactive in time and space.

### Statistics of protein and module variance in supermodules

**Figure 4:**
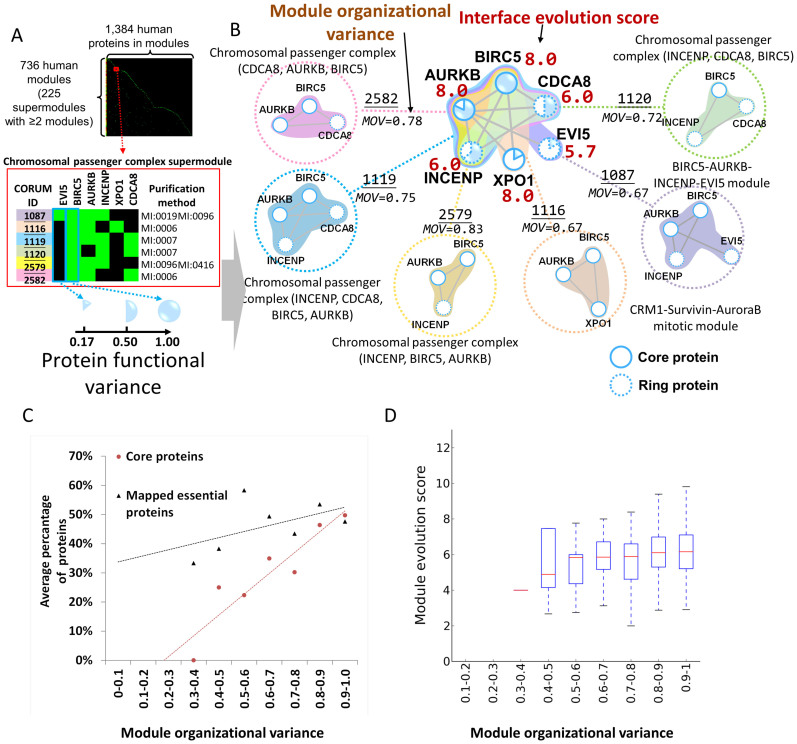
Protein functional variance and module organizational variance of supermodules. (A) Clustering matrix of 736 human modules with 1,384 proteins. The profile of the CPC supermodule with six experimental modules identified by various purification methods. (B) The chromosomal passenger complex supermodule comprises six CORUM modules. Module organizational variance (MOV), protein functional variance (PFV), and IES (red) are shown. Solid and dashed circles denote core proteins and ring proteins, respectively. (C) Pearson's r values between MOV values and percentages of core proteins (red) and mapped essential proteins (black) were 0.93 and 0.52, respectively. (D) The distributions (boxplots) of MOV against module evolution scores (MES).

Proteins often assemble dynamically and cooperate to form the modules that perform biological functions in time and space. Among 1,515 human modules, we found that 1,449 (96%) contain at least one protein that was involved in more than two modules. We iteratively clustered 1,515 human modules into 225 supermodules (including 736 modules) until *J*(A,B) ≤ 0.5 for any pair of modules (Figs. 4A and 4B). Here, we define a supermodule that consists of several modules performing specific biological functions (functional diversities) in different cell states (time) and tissue/cell types (space). We used the functional diversities of a supermodule to understand the characteristics of module organization and variance in PPI networks. Then, we define the functional variance (PFV) of the protein *p* in a supermodule as 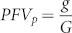, where *g* is the number of the modules in which protein *p* involved and G is the total number of modules of this supermodule. Subsequently, the organizational variance (MOV) of the module *m* in a PPI network is given as 
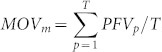
, where *T* is the number of proteins in this module *m*. High MOV implies that the module often plays an important role in a cell and highly involved in various functions and PPI networks in time and space.

Figure 4C shows the correlation of MOV values with percentages of core proteins (Pearson's *r* = 0.93) and mapped essential proteins (Pearson's *r* = 0.52) in modules. For example, three of four proteins (75%) in the CDK1–PCNA–CCNB1–GADD45B module, which has a high MOV value (0.69) in its supermodule, were both core proteins and mapped essential proteins (Fig. 1I). The module evolution scores (MES) increase as MOV values increase up to 0.6 (Fig. 4D), but after that MES values remain ~6. Based on module variance and composition of core/ring components, we observed three factors for this trend: 1) the mean MES values of core-only modules and ring-only modules are 8.03 and 4.81, respectively; 2) the number of core components is often smaller (or similar) than the number of ring components in a module (Fig. 3C); 3) the percentages of core components in modules increase as MOV values increase up to 0.6, but after that percentages of core components remain ~41% (Supplementary Fig. S8). In the CDK1–PCNA–CCNB1 supermodule, MOV values of four modules were ≥0.69 and represented high MES (≥6). In addition, PFV values (median = 0.78) of core proteins were significantly higher (Mann–Whitney *U* test, *P* = 9e-11) than those (median = 0.56) of ring proteins (Supplementary Fig. S9). In the CDK1–PCNA–CCNB1 supermodule, core proteins were involved in multiple modules (PFV ≥ 0.5), whereas ring proteins were not (PFV = 0.25; Fig. 1I).

The chromosomal passenger complex (CPC) supermodule comprises six experimental modules that were derived from various purification methods, including anti bait coimmunoprecipitation (MI:0006), anti tag coimmunoprecipitation (MI:0007), coimmunoprecipitation (MI:0019), pull down (MI:0096), and fluorescence microscopy (MI:0416) (Fig. 4A). This supermodule is organized by six proteins, including aurora-B serine/threonine protein kinase (AURKB), baculoviral IAP repeat-containing protein 5 (BIRC5; survivin), inner centromere protein (INCENP), borealin (CDCA8), ecotropic viral integration site 5 protein homolog (EVI5), and exportin-1 (XPO1/CRM1; Fig. 4B). During early mitosis, CPC is an important mitotic regulatory complex that promotes chromosome alignment by correcting misattachments between chromosomes and microtubules of the mitotic spindle^34^. The CPC supermodule contained the three core proteins BIRC5, AURKB, and XPO1, and the three ring proteins INCENP, CDCA8, and EVI5. In this supermodule, chromosomal passenger complex (INCENP, AURKB, and BIRC5) had the highest MOV value (0.83), and comprised two core proteins and three essential proteins.

Interestingly, the MOV value of the CRM1–Survivin–AuroraB mitotic module (XPO1, BIRC5, and AURKB) was 0.67, and its module evolution score was 8. The core proteins BIRC5 and AURKB were included in most CPC modules (PFV ≥ 0.83), whereas PFV of XPO1 was only 0.17 (Fig. 4B). The functions of the CPC can attribute to the action of the enzymatic core, the AURKB^34^, and the BIRC5 mediates the CPC to target to the centromere and midbody^34^. Previous studies indicate that the BIRC5–XPO1 interaction is essential for CPC localization and activity^35^, implying that XPO1 may play an important role. On the other hand, PFV values of the ring proteins INCENP, CDCA8, and EVI5, were 0.67, 0.5, and 0.17, respectively (Fig. 4B). In human cells, functional CPCs can be targeted, although less efficiently, to centromeres and central spindles in the absence of CDCA8, lack of orthologs in *S. cerevisiae* and *S. pombe*, when BIRC5 is linked covalently to INCENP^34^. During the late stages of mitosis, EVI5 associates with CPC and plays a role in the completion of cytokinesis. Therefore, the present results suggest that the functional variance of core proteins are often significantly higher than those of ring proteins.

### Module variance in different time and space

**Figure 5:**
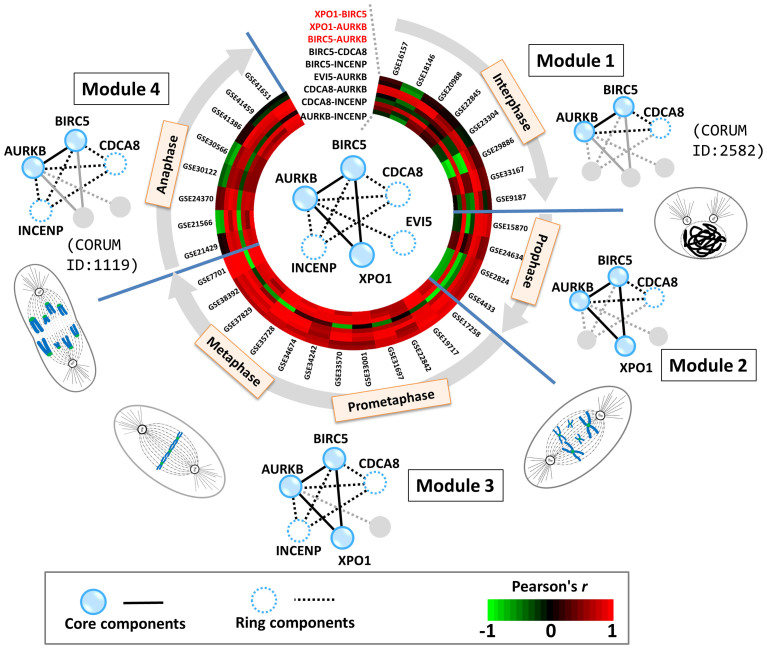
Module and protein variance of chromosomal passenger complex (CPC) supermodule during cell division. Based on 87 gene expression sets, the CPC supermodule, including six genes for BIRC5, AURKB, CDCA8, INCENP, XPO1, and EVI5, consists of 4 modules that were described in more than three gene expression sets. For example, modules 1 and 2 are presented in 8 and 4 sets, respectively. The CPC supermodule can represents the dynamical regulation of cell division in interphase state and mitotic state, comprised of prophase, prometaphase, metaphase, anaphase and telophase (not represented here). Based on gene expression sets, the co-expressions of 9 PPIs in the CPC supermodule are measured using Pearson's *r* values ranging from −1 (green) to 1 (red). Proteins and PPIs with low gene co-expression (Pearson's *r* < 0.5) are shown as gray circles and gray lines. Other proteins and PPIs are indicated with blue circles and black lines. For example, for module 1, Pearson's *r* values of the three PPIs AURKB–BIRC5, AURKB–CDCA8, and BIRC5–CDCA8 are more than 0.5 (red). Solid circles/lines denote core proteins/PPIs, and dashed circles/lines denote ring proteins/PPIs.

**Figure 6:**
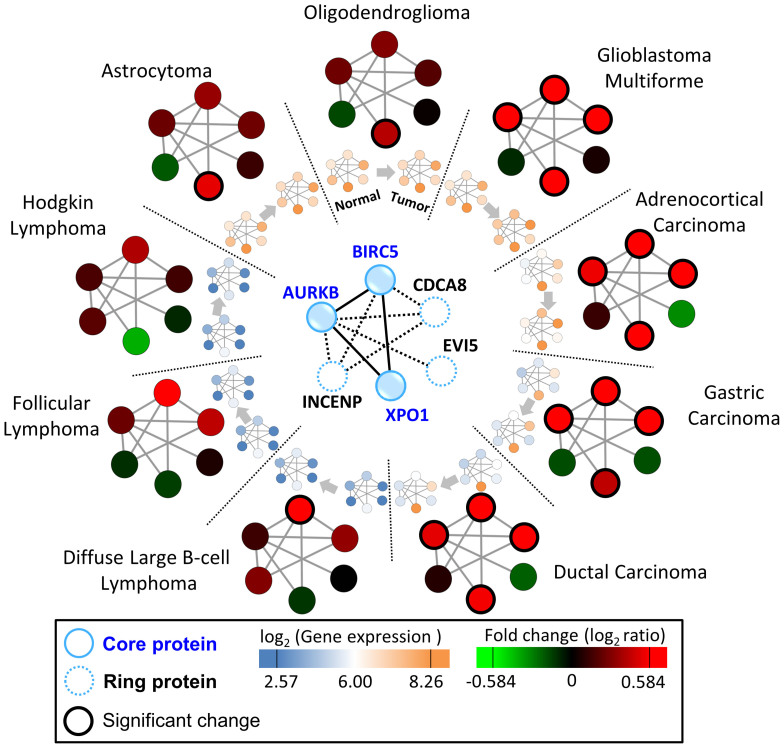
Module variance of chromosomal passenger complex (CPC) supermodule in different tissue and cell types. The gene expression data sets for 9 different tumor types were collected from GEO, and used to identify genes of CPC supermodule with significant expression change between tumor and corresponding normal tissues. In these nine tumor types, the CPC supermodule dynamically assembles its core and ring components to form modules performing specific biological functions during tumorigenesis. High (orange) and low (blue) expression values are scaled by log_2_. Fold change values of these genes for up-regulation (red) and down-regulation (green) are shown in the color scheme. Blue solid circles/lines denote core proteins/PPIs and blue dashed circles/lines denote ring proteins/PPIs. The genes with adjusted *P*-value < 0.05 and fold change >1.3 are considered as significantly changed genes (black thick circle).

Here, we use the CPC supermodule to describe the link from PFV and MOV values to module variance and biological functions in time and space based on 7,208 gene expression data sets (Figs. 5 and 6). To explore the protein and module variance in different cell states, we first utilized CPC supermodule to describe the regulation of cell division (Fig. 5). From 7,208 gene expression sets, we collected 87 sets, which include all 6 proteins and at least one co-expression (Pearson's *r* ≥ 0.5) of interacting protein pairs in CPC supermodule. According to these 87 sets, we derived four modules in CPC supermodule to describe the regulation of cell division in interphase state and mitotic state. The mitotic state is comprised of prophase, prometaphase, metaphase, anaphase and telophase (not represented here), requiring the assembly and disassembly of specific modules within a supermodule. For example, for the module 1 (including three proteins AURKB, BIRC5 and CDCA8) in interphase (Fig. 5), the gene co-expression values of three PPIs (i.e., AURKB–BIRC5, AURKB–CDCA8, and BIRC5–CDCA8) are more than 0.5 in eight sets. Conversely, Pearson's *r* values of the other 6 PPIs (e.g. AURKB–EVI5 and BIRC5–EVI5) in CPC supermodule were less than 0.5.

For the CPC supermodule, we found that the core proteins (e.g. BIRC5 with PFV = 1 and AURKB with PFV = 0.83) of the CPC supermodule play key roles in various biological functions in the regulation of cell division (Fig. 5). AURKB, the enzymatic core of CPC, is activated through binding to BIRC5, and then interacts with CDCA8 to form the module (module 1) in interphase^36^,^37^. Moreover, XPO1 interacts with BIRC5 of the module 1 to form module 2 for tethering the CPC to the centromere in prophase^35^. In prometaphase and metaphase, the AURKB–BIRC5–CDCA8–XPO1 module incorporates INCENP (to form module 3) to promote chromosome alignment^38^. INCENP is a scaffold protein whose N-terminal region can interact with BIRC5 and CDCA8, and C-terminal region can bind to AURKB. Additionally, INCENP localizes the CPC to the central spindle and midbody during anaphase and cytokinesis, respectively^36^. Interestingly, INCENP plays key role in biological functions of CPC, but INCENP is not often co-expressed with AURKB, BIRC5, and CDCA8. In anaphase, XPO1 may dissociate from the module (to form module 4). Finally, EVI5 associates with the CPC and is involved in the completion of cytokinesis^39^. The core proteins (e.g., BIRC5 and AURKB with PFV ≥ 0.83) are co-expressed more frequently than ring proteins (e.g., EVI5 with PFV = 0.17). These results indicate that core and ring components assemble dynamically and cooperate to form the modules for executing specific functions on different time.

To observe the module variance in different tissue and cell types, we collected six gene expression data sets, consisting of tumor and corresponding normal tissue samples in nine tumor types (Fig. 6, Supplementary Table S2, and Text S5). According to gene profiles, these nine tumor types can be simply divided into three groups, including brain (glioblastoma multiforme, oligodendroglioma, and astrocytoma), lymphoma (diffuse large B-cell lymphoma, follicular lymphoma, and Hodgkin lymphoma), and the other (adrenocortical carcinoma, gastric carcinoma, and ductal carcinoma). We employed CPC supermodule on these nine tumor types to observe the module variance. We found that AURKB–BIRC5–CDCA8–XPO1 module is significantly up-regulated (adjusted *P*-value < 0.05 and fold change >1.3) in glioblastoma multiforme, adrenocortical carcinoma, gastric carcinoma, and ductal carcinoma (breast cancer). The dysregulation of the CPC in proliferation has proposed to be associated with aggressive solid tumors^40^. Based on well-known proliferation markers (e.g., MYBL2, BUB1, and PLK1) and cell cycle regulated genes (e.g., CCNE1, CCND1, and CCNB1)^41^, we found that the proliferation markers are indeed only up-regulated in glioblastoma multiforme, adrenocortical carcinoma, gastric carcinoma, and ductal carcinoma. In addition, the gene expression values of CPC supermodule are relatively low (blue) in three lymphoma types with respect to other cancer types and most genes are also non-significantly changed (Fig. 6). These results show that CPC supermodule dynamically assembles its core and ring components to form modules performing specific biological functions during tumorigenesis in these nine tumor types.

### RAD17–RFC-9-1-1 checkpoint module

In this study, we used the RAD17–RFC-9-1-1 checkpoint module (RAD17–RFC-9-1-1 module, CORUM ID: 274) of *H. sapiens* to describe module organization and variance in PPI networks. This module comprises 16 PPIs and 8 proteins (Supplementary Fig. S10A), including the cell cycle checkpoint proteins RAD1/RAD9A/RAD17 (RAD1/RAD9A/RAD17), the replication factor C subunits 2/3/4/5 (RFC2/RFC3/RFC4/RFC5), and checkpoint protein HUS1 (HUS1). During the cell cycle, the RAD17–RFC-9-1-1 module is involved in the early steps of the DNA damage checkpoint response^42^. Using the RAD17–RFC-9-1-1 module in *H. sapiens* as a module template, homologous modules across 127 species and 5 taxonomic divisions were all found to regulate DNA damage recognition (Supplementary Fig. S10B). The ten PPI families (e.g. RFC2–RFC5, RAD17–RFC4, and RFC3–RFC4) and the six PPI families (e.g. HUS1–RAD9A and HUS1–RAD1) of this module were regarded as core components and ring components (Supplementary Fig. S10C), respectively.

Five core proteins RFC2 (degree = 23), RFC3 (degree = 13), RFC4 (degree = 17), and RFC5 (degree = 13) were determined as hubs (degree ≥ 10) in the human PPI network (Supplementary Fig. S10D). Conversely, the degree of all ring proteins (HUS1, RAD1, and RAD9A) was 4. In addition, the core proteins, RFC2, RFC3, RFC4, RFC5, and RAD17, were homologous to essential proteins recorded in DEG (Supplementary Fig. S10E) and annotated with several essential GO MF terms, such as “DNA clamp loader activity” and “nucleoside-triphosphatase activity.” During DNA replication, RFC binds to primed templates and recruits PCNA to the site of replication^43^. In addition, RAD17 associates with these four small RFC subunits and forms an RFC-like complex that acts as a DNA damage sensor^42^. Therefore, the present results suggest that core proteins of RFC subunits and RAD17 are essential in the RAD17–RFC-9-1-1 module.

Among collected 7,208 gene expression data sets of *H. sapiens*, 309 contained at least one co-expression of interacting protein pairs in the RAD17–RFC-9-1-1 module with Pearson's *r* values of ≥0.5. Among these 309 sets, CEs of 10 core PPIs were significantly higher than those of the three ring PPIs (Supplementary Fig. S10F). For example, CE of the interaction proteins, RFC2 and RFC5, was 0.74, with Pearson's *r* values of ≥0.5 in 229 gene expression sets among 309 sets. The ring proteins RAD9A, RAD1, and HUS1 of the RAD17–RFC-9-1-1 module form a PCNA-like ring structure that may interact with RFC-like complexes to regulate DNA binding in ATP-dependent or ATP-independent manners^42^.

The RAD17–RFC-9-1-1 supermodule comprises the RFC2–5 module (CORUM ID: 2200), the RAD17–RFC module (CORUM ID: 270), and the RAD17–RFC-9-1-1 module (CORUM ID: 274). The core proteins (i.e. RFC2, RFC3, RFC4, and RFC5) with PFV values of 1 were consistently involved in these three modules to perform various biological functions (Supplementary Fig. S10G). Conversely, the PFV value of the three ring proteins was 0.33, and these are included in one module to perform one of functions of the RAD17-RFC-9-1-1 supermodule. Moreover, MOV values of RAD17–RFC-9-1-1, RAD17–RFC, and RFC2–5 modules were 0.71, 0.93, and 1.0, respectively, and were highly correlated with MES (7.32, 8.99, and 9.81, respectively).

**Figure 7:**
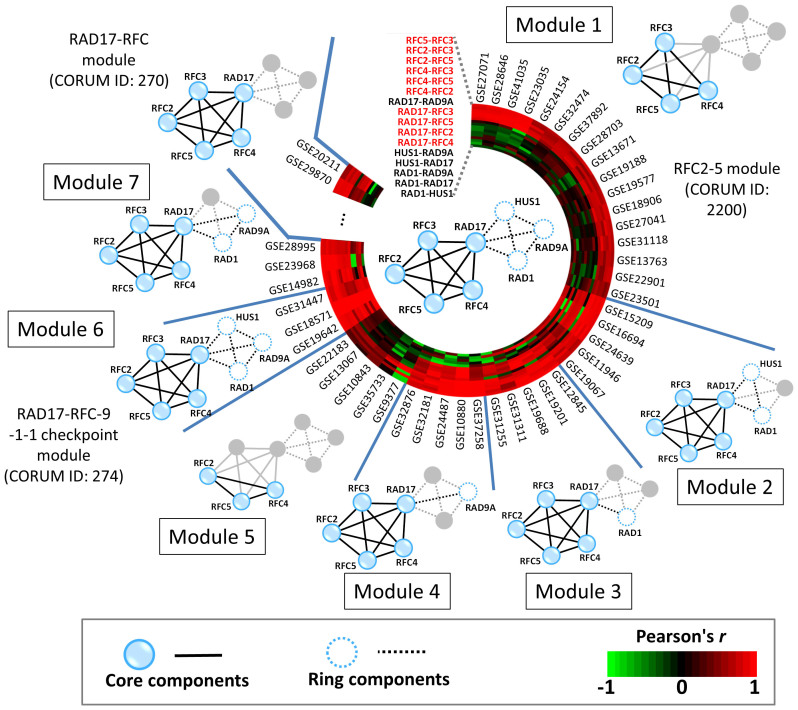
Module and protein variance of the RAD17–RFC-9-1-1 supermodule during DNA replication. Among 309 gene expression sets, the RAD17–RFC-9-1-1 supermodule includes seven modules with ≥3 gene expression sets containing all genes for RFC2–5, RAD17, RAD1, RAD9A, and HUS1. Among these seven modules, only two (modules 1 and 6) are recorded in the CORUM database. The module variance provides the clues for DNA damage sensors during DNA replication in an ATP-dependent manner.

Figure 7 shows the module variance of the RAD17–RFC-9-1-1 supermodule during DNA replication from 309 gene expression sets, which are recorded in the GEO database and include all 8 proteins of this supermodule. Based on these 309 gene expression sets, we inferred seven modules that were described in more than three gene expression sets. Among these seven inferred modules, the RFC2-5 module (module 1, CORUM ID: 2200) and the RAD17–RFC-9-1-1 module (module 6, CORUM ID: 274) were recorded in the CORUM database and were derived from 17 and 5 gene expression sets, respectively. Inferred module 5, namely the RAF2–RAF4–RAF5 module, has been studied for DNA-dependent ATPase activity stimulated by PCNA (similar to the five-subunit RFC) and can unload PCNA from singly nicked circular DNA^44^. In addition, we found that the RAD17–RFC module included the five proteins (i.e., RAD17 and RFC2-5), and interacts with RAD1 to form module 3, with RAD9A to form module 4, with RAD1 and HUS1 to form module 2, and with RAD9A and RAD1 to form module 7 for the regulation of DNA damage checkpoint response^42^. Interestingly, according to these 309 sets, the RAD17–RFC module did not interact with HUS1 to form a module, and this was in agreement with a previous study^42^. During DNA replication, the RFC2-5 module (module 1) and the RFC2–RFC4–RFC5 module (module 5) possess DNA-dependent ATPase activity and are not responsive to the addition of PCNA^45^ (Fig. 7). In the early steps of DNA damage recognition, the RAD17–RFC module (CORUM ID: 270) activates the checkpoint response^46^, and then binds to nicked circular, gapped, and primed DNA to recruit the RAD9A–RAD1–HUS1 module (module 6; CORUM ID: 274) for ATP-dependent DNA damage sensor^42^. These results indicate that RFC2, RFC3, RFC4, and RFC5 play major roles in DNA damage recognition and that the RAD9, RAD1, and HUS1 could regulate them to bind to DNA with or without ATP. Interestingly, the core protein RAD17 forms the bridge between core and ring components, and co-expressions of the three core PPIs (i.e., RFC2-RAD17, RFC3-RAD17 and RFC4-RAD17) are slightly lower than those of the other core PPIs.

### Conclusions

We have analyzed network topology, gene essentiality, protein/module variance, and gene co-expression to summarize the observations of module organization and variance in the following: 1) a module comprises core and ring components and the former is more conserved and essential during organizational changes in different biological states or conditions; 2) core components often perform the major biological functions of a module, whereas the ring components are indirectly involved in biological functions through collaborations with core components.

## Methods

### Homologous modules

Here, we used the module template M (including proteins A, B, C, and D) with six interfaces A–B, A–C, A–D, B–C, B–D, and C–D as an example (Fig. 1), and the homologous module of M was defined as follows: 1) A′, B′, C′, and D′ are homologous proteins of A, B, C, and D, respectively, with statistically significant sequence similarities (BLASTP *E*-values ≤ 10^−10^)^47^,^48^; 2) A′–B′, A′–C′, A′–D′, B′–C′, B′–D′, and C′–D′ are the best-matching homologous PPIs of A–B, A–C, A–D, B–C, B–D, and C–D, respectively, with statistically significant joint sequence similarities (joint *E*-value ≤ 10^−40^)^14^; 3) A′, B′, C′, and D′ are the homologous module of template M, as indicated by high topological similarity (protein-aligned ratio of ≥0.5 and PPI-aligned ratio of ≥0.3). Protein- and PPI-aligned ratios were defined as the number of proteins and PPIs in the homologous module divided by the number of proteins and PPIs in the module template, respectively. Protein-aligned ratios of ≥0.5 and PPI-aligned ratios of ≥0.3 indicated topological similarity according to statistical analyses of 37,197 structural modules (187 reference modules) in 1,442 species based on the KEGG MODULE database^49^ (Supplementary Fig. S1).

### PPI evolution score and protein interface evolution score

We propose the PPI evolution score (PPIES) and protein interface evolution score (IES) to identify core and ring components of a module. To compute the PPIES of a PPI in a module family, we clustered NCBI taxonomy^24^ into six taxonomic divisions: mammals (MAM), vertebrates (VRT), invertebrates (INV), plants (PLN), bacteria (BCT), and archaea (ARC) (Supplementary Table S3). For each PPI *z* of a module family, PPIES was defined as
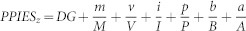
where *DG* is the number of taxonomic divisions that contain at least one species in homologous PPIs of the PPI *z* (Fig. 1D); *M*, *V*, *I*, *P*, *B*, and *A* are the total numbers of species of homologous modules belonging to MAM, VRT, INV, PLN, BCT, and ARC, in the module family, and *m*, *v*, *i*, *p*, *b*, and *a* are the numbers of species belonging to their respective taxonomic divisions of homologous PPIs of the PPI *z*, respectively (Fig. 1E). For each protein *k* in a module family, IES was set to the maximum PPIES, and was defined as 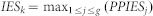, where *g* is the number of proteins that interact with protein *k*. Here, we considered proteins with IES ≥ 7 and PPIs with PPIES ≥ 7 as core components of a module; and all other proteins and PPIs were considered ring components. To evaluate conservation of modules during evolution for each module *d* in a module family, module evolution score (MES) is set to the mean PPIES and is defined as 
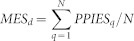
, where *N* is the number of PPIs within module *d*.

### Supermodule

In the present study, supermodules comprised several modules, often with specific biological functions, and their functional diversity was defined by numbers of modules. Initially, 1,515 human CORUM modules were clustered into supermodules using the Jaccard similarity coefficient *J*(A,B)^50^. The *J*(A,B) is defined as 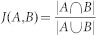, where *A* ∩ *B* is the number of common proteins (intersection set) in modules A and B, and *A* ∪ *B* is the number of the union protein set in modules A and B. Here, modules A and B are clustered into one group if *J*(A,B) ≥ 0.5, and the ordering for adding modules is based on the module size (the largest one has the highest priority). Based on this threshold, we iteratively clustered modules and groups into supermodules until *J*(A,B) ≤ 0.5 for any pair of modules (or groups). Finally, we clustered 1,515 modules into 252 supermodules (including 736 modules) and 115 supermodules (including 462 modules) when the numbers of modules in a supermodule are more than 2 and 3 modules, respectively. Specifically, the CDK1–PCNA–CCNB1–GADD45B module was grouped with 3 other experimental modules to form the CDK1–PCNA–CCNB1 supermodule, which included RalBP1–CDK1–CCNB1, CDK1–CCNB1–PTCH1, and CDK1–PCNA–CCNB1–GADD45A modules (Fig. 1G). The functional diversity of the CDK1–PCNA–CCNB1 supermodule was 4.

### Protein-protein interactions in gene expression profiles

Proteins and PPIs change over time to assemble and disassemble a module for executing biological processes. Here, we quantified the variance of proteins and PPIs in time and space by assessing correlations between expression profiles of interacting proteins in 7,208 gene expression data sets (≥3 samples) derived from GEO^16^ (Supplementary Fig. S7). To avoid the influence of genes with low expression and variance, we selected the gene *j* in a gene expression set based on the following criteria: average expression (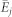) ≥ to the mean expression of all genes (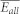) in a gene expression set; or the standard deviation of expression (S*_j_*) ≥ to the standard deviation of expression values for all genes (S*_all_*) in the gene expression set. For each module, we collected expression profiles contained expression values of all proteins in this module, and then calculated Pearson's *r* values for each PPI within the module to construct correlation matrix. Here, we assume that an active module performed biological functions in a cell if at least one PPI of the module had high Pearson's r ≥ *h* (here, *h* was set at 0.3, 0.5, or 0.7). For a PPI *p* (proteins *i* and *j*) in an active module, the co-expression ratio (CE) at the threshold *h* is defined as 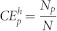, where *N* is the total number of these 7,208 expression profiles with at least one high co-expression (Pearson's *r* ≥ *h*) of any PPI of this module; and *N_p_* is the number of expression profiles containing high co-expression of proteins *i* and *j* with Pearson's *r* values of ≥*h*. For example, the CE of CDK1–CCNB1 is 0.76, reflecting high co-expression (Pearson's *r* ≥ 0.5) in 825 of 1,085 gene expression sets when *h* = 0.5 (Fig. 1I).

## Author Contributions

C.Y.L., Y.S.L. and J.M.Y. conceived and designed the experiments. T.L.L., Y.W.L. and J.M.Y. implemented the program. C.Y.L., T.L.L., Y.W.L. and J.M.Y. performed the experiments and analyzed the data. C.Y.L., T.L.L., Y.Y.C., C.T.L. and J.M.Y. wrote the paper.

## Supplementary Material

Supplementary Information Supplementary information

## Figures and Tables

**Table 1:** Summary of interface evolution, orthology, and essential molecular functions

Interface evolution score	Number of total proteins	Number of annotated proteins (recorded as essential genes)	Number of unannotated proteins	Orthologs (PORC)^a^	181 essential GO MF terms^b^	Children of 181 essential GO MF terms^c^	Total
≥11	115	114 (99%)	1	0	0	1	1 (100%)
≥9	306	269 (88%)	37	14	10	19	28 (76%)
≥7	3381	2169 (64%)	1212	152	368	303	599 (49%)
<7	4569	1459 (32%)	3110	217	491	689	1175 (38%)

^a^The number of proteins contained at least an orthologous protein, recorded in the PORC orthology database^23^, of essential proteins.

^b^The number of proteins contained at least an essential GO MF terms.

^c^The number of proteins contained at least a child term of 181 essential GO MF terms.

## References

[b1] Gavin A. C. *et al.* Proteome survey reveals modularity of the yeast cell machinery. Nature 440, 631–636 (2006). 16429126 10.1038/nature04532

[b2] Segal E. *et al.* Module networks: identifying regulatory modules and their condition-specific regulators from gene expression data. Nat. Genet. 34, 166–176 (2003). 12740579 10.1038/ng1165

[b3] Wagner G. P., Pavlicev M. & Cheverud J. M. The road to modularity. Nat. Rev. Genet. 8, 921–931 (2007). 18007649 10.1038/nrg2267

[b4] Ruepp A. *et al.* CORUM: the comprehensive resource of mammalian protein complexes. Nucleic Acids Res. 36, D646–650 (2008). 17965090 10.1093/nar/gkm936 PMC2238909

[b5] Bader G. D. & Hogue C. W. An automated method for finding molecular complexes in large protein interaction networks. BMC Bioinformatics 4, 2 (2003). 12525261 10.1186/1471-2105-4-2 PMC149346

[b6] Nepusz T., Yu H. & Paccanaro A. Detecting overlapping protein complexes in protein-protein interaction networks. Nat. Methods 9, 471–472 (2012). 22426491 10.1038/nmeth.1938 PMC3543700

[b7] Ideker T., Ozier O., Schwikowski B. & Siegel A. F. Discovering regulatory and signalling circuits in molecular interaction networks. Bioinformatics 18 Suppl 1, S233–240 (2002). 12169552 10.1093/bioinformatics/18.suppl_1.s233

[b8] Yamada T., Kanehisa M. & Goto S. Extraction of phylogenetic network modules from the metabolic network. BMC Bioinformatics 7, 130 (2006). 16533389 10.1186/1471-2105-7-130 PMC1501048

[b9] Snel B. & Huynen M. A. Quantifying modularity in the evolution of biomolecular systems. Genome Res. 14, 391–397 (2004). 14993205 10.1101/gr.1969504 PMC353226

[b10] Campillos M., von Mering C., Jensen L. J. & Bork P. Identification and analysis of evolutionarily cohesive functional modules in protein networks. Genome Res. 16, 374–382 (2006). 16449501 10.1101/gr.4336406 PMC1415216

[b11] Levy E. D. & Pereira-Leal J. B. Evolution and dynamics of protein interactions and networks. Curr. Opin. Struct. Biol. 18, 349–357 (2008). 18448325 10.1016/j.sbi.2008.03.003

[b12] Ettema T., van der Oost J. & Huynen M. Modularity in the gain and loss of genes: applications for function prediction. Trends Genet. 17, 485–487 (2001). 11525815 10.1016/s0168-9525(01)02384-8

[b13] Chen Y. C., Lo Y. S., Hsu W. C. & Yang J. M. 3D-partner: a web server to infer interacting partners and binding models. Nucleic Acids Res. 35, W561–567 (2007). 17517763 10.1093/nar/gkm346 PMC1933210

[b14] Chen C. C., Lin C. Y., Lo Y. S. & Yang J. M. PPISearch: a web server for searching homologous protein-protein interactions across multiple species. Nucleic Acids Res. 37, W369–375 (2009). 19417070 10.1093/nar/gkp309 PMC2703927

[b15] Lin C. Y., Lin Y. W., Yu S. W., Lo Y. S. & Yang J. M. MoNetFamily: a web server to infer homologous modules and module-module interaction networks in vertebrates. Nucleic Acids Res. 40, W263–270 (2012). 22689643 10.1093/nar/gks541 PMC3394321

[b16] Barrett T. *et al.* NCBI GEO: archive for high-throughput functional genomic data. Nucleic Acids Res. 37, D885–890 (2009). 18940857 10.1093/nar/gkn764 PMC2686538

[b17] Aranda B. *et al.* The IntAct molecular interaction database in 2010. Nucleic Acids Res. 38, D525–531 (2010). 19850723 10.1093/nar/gkp878 PMC2808934

[b18] Stark C. *et al.* The BioGRID Interaction Database: 2011 update. Nucleic Acids Res. 39, D698–704 (2011). 21071413 10.1093/nar/gkq1116 PMC3013707

[b19] Xenarios I. *et al.* DIP, the Database of Interacting Proteins: a research tool for studying cellular networks of protein interactions. Nucleic Acids Res. 30, 303–305 (2002). 11752321 10.1093/nar/30.1.303 PMC99070

[b20] Mewes H. W. *et al.* MIPS: analysis and annotation of genome information in 2007. Nucleic Acids Res. 36, D196–201 (2008). 18158298 10.1093/nar/gkm980 PMC2238900

[b21] Ceol A. *et al.* MINT, the molecular interaction database: 2009 update. Nucleic Acids Res. 38, D532–539 (2010). 19897547 10.1093/nar/gkp983 PMC2808973

[b22] Lo Y. S., Chen Y. C. & Yang J. M. 3D-interologs: an evolution database of physical protein- protein interactions across multiple genomes. BMC Genomics 11 Suppl 3, S7 (2010). 21143789 10.1186/1471-2164-11-S3-S7 PMC2999352

[b23] Kersey P. *et al.* Integr8 and Genome Reviews: integrated views of complete genomes and proteomes. Nucleic Acids Res. 33, D297–302 (2005). 15608201 10.1093/nar/gki039 PMC539993

[b24] Sayers E. W. *et al.* Database resources of the National Center for Biotechnology Information. Nucleic Acids Res. 39, D38–51 (2011). 21097890 10.1093/nar/gkq1172 PMC3013733

[b25] Vairapandi M., Balliet A. G., Hoffman B. & Liebermann D. A. GADD45b and GADD45g are cdc2/cyclinB1 kinase inhibitors with a role in S and G2/M cell cycle checkpoints induced by genotoxic stress. J. Cell. Physiol. 192, 327–338 (2002). 12124778 10.1002/jcp.10140

[b26] Lindqvist A., van Zon W., Karlsson Rosenthal C. & Wolthuis R. M. Cyclin B1-Cdk1 activation continues after centrosome separation to control mitotic progression. PLoS Biol. 5, e123 (2007). 17472438 10.1371/journal.pbio.0050123 PMC1858714

[b27] Barabasi A. L. & Oltvai Z. N. Network biology: understanding the cell's functional organization. Nat. Rev. Genet. 5, 101–113 (2004). 14735121 10.1038/nrg1272

[b28] Seyed-Allaei H., Bianconi G. & Marsili M. Scale-free networks with an exponent less than two. Phys. Rev. E Stat. Nonlin. Soft Matter Phys. 73, 046113 (2006). 16711884 10.1103/PhysRevE.73.046113

[b29] D'Antonio M. & Ciccarelli F. D. Modification of gene duplicability during the evolution of protein interaction network. PLoS Comput. Biol. 7, e1002029 (2011). 21490719 10.1371/journal.pcbi.1002029 PMC3072358

[b30] Peregrin-Alvarez J. M., Sanford C. & Parkinson J. The conservation and evolutionary modularity of metabolism. Genome Biol. 10, R63 (2009). 19523219 10.1186/gb-2009-10-6-r63 PMC2718497

[b31] Kobayashi K. *et al.* Essential Bacillus subtilis genes. Proc. Natl. Acad. Sci. U. S. A. 100, 4678–4683 (2003). 12682299 10.1073/pnas.0730515100 PMC153615

[b32] Zhang R. & Lin Y. DEG 5.0, a database of essential genes in both prokaryotes and eukaryotes. Nucleic Acids Res. 37, D455–458 (2009). 18974178 10.1093/nar/gkn858 PMC2686491

[b33] Carter S. L., Brechbuhler C. M., Griffin M. & Bond A. T. Gene co-expression network topology provides a framework for molecular characterization of cellular state. Bioinformatics 20, 2242–2250 (2004). 15130938 10.1093/bioinformatics/bth234

[b34] Vader G., Kauw J. J., Medema R. H. & Lens S. M. Survivin mediates targeting of the chromosomal passenger complex to the centromere and midbody. EMBO Rep 7, 85–92 (2006). 16239925 10.1038/sj.embor.7400562 PMC1369225

[b35] Knauer S. K., Bier C., Habtemichael N. & Stauber R. H. The Survivin-Crm1 interaction is essential for chromosomal passenger complex localization and function. EMBO Rep 7, 1259–1265 (2006). 17099693 10.1038/sj.embor.7400824 PMC1794705

[b36] van der Horst A. & Lens S. M. Cell division: control of the chromosomal passenger complex in time and space. Chromosoma 123, 25–42 (2014). 24091645 10.1007/s00412-013-0437-6 PMC3967068

[b37] Gassmann R. *et al.* Borealin: a novel chromosomal passenger required for stability of the bipolar mitotic spindle. J. Cell Biol. 166, 179–191 (2004). 15249581 10.1083/jcb.200404001 PMC2172304

[b38] Sampath S. C. *et al.* The chromosomal passenger complex is required for chromatin-induced microtubule stabilization and spindle assembly. Cell 118, 187–202 (2004). 15260989 10.1016/j.cell.2004.06.026

[b39] Faitar S. L., Sossey-Alaoui K., Ranalli T. A. & Cowell J. K. EVI5 protein associates with the INCENP-aurora B kinase-survivin chromosomal passenger complex and is involved in the completion of cytokinesis. Exp. Cell Res. 312, 2325–2335 (2006). 16764853 10.1016/j.yexcr.2006.03.032

[b40] Gerlach U. *et al.* Centrosome-, chromosomal-passenger- and cell-cycle-associated mRNAs are differentially regulated in the development of sporadic colorectal cancer. J. Pathol. 208, 462–472 (2006). 16402339 10.1002/path.1914

[b41] Whitfield M. L., George L. K., Grant G. D. & Perou C. M. Common markers of proliferation. Nat. Rev. Cancer 6, 99–106 (2006). 16491069 10.1038/nrc1802

[b42] Bermudez V. P. *et al.* Loading of the human 9-1-1 checkpoint complex onto DNA by the checkpoint clamp loader hRad17-replication factor C complex in vitro. Proc. Natl. Acad. Sci. U. S. A. 100, 1633–1638 (2003). 12578958 10.1073/pnas.0437927100 PMC149884

[b43] Waga S. & Stillman B. The DNA replication fork in eukaryotic cells. Annu. Rev. Biochem. 67, 721–751 (1998). 9759502 10.1146/annurev.biochem.67.1.721

[b44] Cai J. *et al.* A complex consisting of human replication factor C p40, p37, and p36 subunits is a DNA-dependent ATPase and an intermediate in the assembly of the holoenzyme. J. Biol. Chem. 272, 18974–18981 (1997). 9228079 10.1074/jbc.272.30.18974

[b45] Ellison V. & Stillman B. Reconstitution of recombinant human replication factor C (RFC) and identification of an RFC subcomplex possessing DNA-dependent ATPase activity. J. Biol. Chem. 273, 5979–5987 (1998). 9488738 10.1074/jbc.273.10.5979

[b46] Lindsey-Boltz L. A., Bermudez V. P., Hurwitz J. & Sancar A. Purification and characterization of human DNA damage checkpoint Rad complexes. Proc. Natl. Acad. Sci. U. S. A. 98, 11236–11241 (2001). 11572977 10.1073/pnas.201373498 PMC58713

[b47] Matthews L. R. *et al.* Identification of potential interaction networks using sequence-based searches for conserved protein-protein interactions or “interologs”. Genome Res. 11, 2120–2126 (2001). 11731503 10.1101/gr.205301 PMC311221

[b48] Yu H. *et al.* Annotation transfer between genomes: protein-protein interologs and protein-DNA regulogs. Genome Res. 14, 1107–1118 (2004). 15173116 10.1101/gr.1774904 PMC419789

[b49] Kanehisa M. *et al.* KEGG for linking genomes to life and the environment. Nucleic Acids Res. 36, D480–484 (2008). 18077471 10.1093/nar/gkm882 PMC2238879

[b50] Willett P., Barnard J. M. & Downs G. M. Chemical similarity searching. J. Chem. Inf. Comput. Sci. 38, 983–996 (1998).

