# Supplementary material for: Module organization and variance in protein-protein interaction networks
Source: Sci Rep. 2015 Mar 23;5:9386. doi: 10.1038/srep09386 (PMC4369690; doi:10.1038/srep09386)
Supplement: Supplementary Information [file srep09386-s1.pdf]

# Module organization and variance in protein-protein interaction networks

Chun-Yu Lin[1], Tsai-Ling Lee[1], Yi-Yuan Chiu[1], Yi-Wei Lin[1], Yu-Shu Lo[1], Chih-Ta Lin[1], and Jinn-Moon Yang[1,2*]

[1]Institute of Bioinformatics and Systems Biology, National Chiao Tung University, Hsinchu, Taiwan

[2]Department of Biological Science and Technology, National Chiao Tung University, Hsinchu, Taiwan

***Corresponding author**

Contact information:

Address: 75 PO-Ai Street, Hsinchu, Taiwan, 30050

Tel: 886-3-5712121 ext.56942; Fax: 886-3-5729288

E-mail: moon@faculty.nctu.edu.tw

**Supplementary Text 1: Homologous modules and human PPI network**

To observe topologies and functional similarities of homologous modules in module families, we collected 37,197 structural modules, from 187 reference modules across 1,442 organisms from the KEGG MODULE database[1]. According to the data set, protein-aligned ratios of 96% (35,820) between homologous and their reference modules were more than 0.5 (Supplementary Fig. S1A). To determine topological similarity thresholds between reference and its homologous modules, we added intra-module PPIs using the following PPI databases: 1) 461,077 experimental PPIs from annotated PPI databases, including IntAct[2], BioGRID[3], DIP[4], MIPS[5], and MINT[6]; 2) sequence-based homologous PPIs with joint *E*-values of $\leq 10^{-40}$ [7] among 461,077 experimental PPIs; and 3) 86,252 structure-based homologous PPIs with *Z*-scores of $\geq 4$[8]. Among 37,197 organism-specific structural modules, we added at least one PPI for 10,729 modules, and 80% PPI-aligned ratios between reference modules and their homologous modules were $\geq 0.3$ (Supplementary Fig. S1B). Here, we set the protein-aligned ratio and PPI-aligned ratio to 0.5 and 0.3, respectively, to identify homologous modules of a module template.

**Supplementary Text 2: connectivity of modules**

A module is relatively autonomous and often has high connectivity ($C_t$) within a PPI network. To observe connectivity ($C_t$) of a module in a PPI network, we quantified the connectivity by $C_t = \frac{m}{C_2^n}$ [9], where $n$ and $m$ are the numbers of connected proteins and PPIs in a module. A $C_t$ value of 1 indicates that proteins are completely interconnected in a module. For $C_t$ of core (or ring) components, $n$ and $m$ are the numbers of connected core (or ring) proteins and PPIs in a module. In this study, $C_t$ of core (or ring) components were evaluated while $n$ is larger than 3. Here, we computed $C_t$ of modules using the human PPI network. Supplementary Fig. S2A shows the $C_t$ of core and ring components, module templates, and their respective extended modules. Extended modules were extended by one-layer of PPIs and proteins in the module template (M). We assume that the module M consists of a set (P) of proteins and a set (I) of protein-protein interactions (PPIs). The one-layer-extended module of this module M includes a set (P∪P') of proteins and a set (I') of PPIs, where P' consists of the interacting proteins of each protein in set P; I' consists of the PPIs of the proteins in the set P∪P'.

Among 1,519 module templates, $C_t$ values of more than 0.6 were observed in 71% (1,081) of cases. In contrast, $C_t$ values were more than 0.6 for only 5% (71) of extended modules. Moreover, 90% of core components and 81% of ring components had $C_t$ values of ≥0.6. Similarly, 58,041 modules that were homologous to module templates had $C_t$ values of ≥0.6 in 76% of cases (44,319), whereas only 1% (842) of their extended modules had $C_t$ values of ≥0.6 (Supplementary Fig. S3A). These results indicate that core components have the highest connectivity, and that the modules also have high connectivity.

**Supplementary Text 3: biological functions of modules**

Through assembly and cooperation of proteins in a PPI network, components of a module simultaneously perform certain biological functions. Based on the relative specificity similarity (RSS) [10] of GO terms, such as biological process (BP) and cellular component (CC), we computed AvgRSS scores to assess shared biological functions of all protein pairs in a module. The AvgRSS is defined as $AvgRSS = \sum_{i=1}^{n}\sum_{j=1}^{n} \mathrm{RSS(i,\ j)} \Big/ C_2^n, i \neq j$, where $i$ and $j$ are any two proteins of a module and $n$ is the number of proteins in the module.

To elucidate biological functions of modules, we compared module templates, their core and ring components, and their extended modules. For 1,519 module templates, BP and CC AvgRSS scores were more than 0.6 in 89% and 97% of cases, respectively (Supplementary Figs. S2B and S2C), and these scores were significantly higher than those of extended modules (Mann–Whitney U test, $P \cong 0$). In addition, BP and CC average AvgRSS scores of core components were higher than others, including ring components (Mann–Whitney $U$ test, $P$ =2e-7 for BP; $P$ =2e-21 for CC), whole module templates ($P$ =1e-7 for BP; $P$ =1e-14 for CC), and extended modules ($P$ =3e-239 for BP; $P$ =5e-262 for CC). CC AvgRSS scores (97%) of templates were slightly higher than those of their ring components (94%) with AvgRSS scores of ≥0.6. Furthermore, BP and CC AvgRSS scores were more than 0.6 for 81% and 94% of homologous modules, respectively (Supplementary Figs. S3B and S3C). Similarly, BP and CC average AvgRSS scores for core components of homologous modules were also significantly higher than those of ring components ($P$ =0.0036 for BP; $P$ =3e-16 for CC). For example, BP and CC AvgRSS scores for the CDC2–PCNA–CCNB1–GADD45B homologous module in *H. sapiens* were 0.79 and 0.84, but for extended modules they were only 0.43 and 0.25, respectively. The core components of this module had high BP and CC AvgRSS scores of 0.89 and 0.85, respectively. These results indicate that homologous modules of a template have highly similar biological functions and that their core components regulate similar biological processes and are often localized to the same cellular compartment.

**Supplementary Text 4: GO term analysis of essential proteins**

GO terms provide the descriptions of the biological process (BP), cellular component (CC), and molecular function (MF) of a protein[11]. According to a modified term frequency-inverse document frequency (*TF-IDF*) scoring scheme[12], we identified 160 essential MF terms that describe the functional relationships of essential proteins and core proteins of the module families (Supplementary Table S1). First, we collected 8,364 essential proteins, called EP8364, from the DEG database and 160,598 proteins, called CG27, over 27 completed genomes. The proteins in these two sets contained at least one GO MF or GO BP terms. The "occurrence ratio" ($CR_t$) of a GO MF term ($t$) was defined as $CR_t = P_t/T$, where $P_t$ is the number of proteins with term $t$, and $T$ is the total number of proteins in the given set. For example, the occurrence ratio of the term "rRNA binding" was 0.0497 in the EP8364 set for $P_t$ = 416 and $T$ = 8,364. The distribution of the occurrence ratios of 1,886 GO MF terms between core proteins and essential proteins was similar (Pearson's $r$ = 0.86). In contrast, Pearson's $r$ for 4,699 GO BP terms was 0.27, because BP terms often describe a series of events accomplished by one or more ordered assemblies of molecular functions. The MF and BP terms are suitable for analyses at the protein and module levels, respectively.

Next, we developed a "unique ratio" ($UR$) to statistically measure the importance of the GO MF term (i.e., specificity of a protein) by a modified *TF-IDF* scoring scheme[12]. The unique ratio of a GO MF term $t$ was defined as $UR_t = CR_t^{EP}/CR_t^{CG}$, where $CR_t^{EP}$ and $CR_t^{CG}$ are the occurrence ratios of term $t$ in sets EP8364 and CG27, respectively. For example, the unique ratio of the term "rRNA binding" was determined as 9.72 for $CR_t^{EP}$ = 0.0497 and $CR_t^{CG}$ =0.0051. Finally, we selected 160 essential GO MF terms that are statistically significant specificity to essential proteins with $UR \geq 2$ and $p$-value $\leq 0.05$ (hypergeometric distribution). We discarded the terms of specific species (e.g., "azobenzene reductase activity") and those with high usage but without the specificity (e.g., "protein binding").

To analyze the characteristics and functions of the core components, we clustered these 160 essential GO MF terms into 12 groups: Translation (30 terms, 17%), Transcription (9 terms, 5%), Carbohydrate metabolism (22 terms, 12%), Lipid metabolism (11 terms, 6%), Amino acid metabolism (10 terms, 6%), DNA replication (11 terms, 6%), RNA degradation (6 terms, 3%), Purine metabolism (11 terms, 6%), Pyrimidine metabolism (4 terms, 2%), Cell cycle (4 terms, 2%), Oxidative phosphorylation (5 terms, 3%) (Supplementary Fig. S4A and Table S1). The largest percentage (17%) of the essential GO MF terms was assigned to Translation, including such terms as "rRNA binding" ($UR$ = 9.72), "translation release factor activity, codon specific" ($UR$ = 6.48),

"structural constituent of ribosome" (*UR* = 4.72), and "tRNA binding" (*UR* = 8.38). In the process of transcription, the information contained in a segment of DNA is transferred to a newly assembled piece of mRNA. The central dogma of molecular biology, including DNA replication, transcription, and translation, is the fundamental of life for sequence information transfer[13]. Among the 160 essential GO MF terms, 31% of essential GO MF terms were involved in the central dogma (Supplementary Fig. S4A). Furthermore, we also analyzed the percentage of GO MF groups in 3,366 essential proteins (Supplementary Fig. S4B). Seventy-two percent of the essential proteins were annotated with GO MF terms that were related to the central dogma, such as "translation" (55%).

Among the 160 essential GO MF terms, 33 terms (21%; e.g., "acetyl-CoA carboxylase activity", *UR* = 9.25) were recorded for Carbohydrate and Lipid metabolisms, which mediate the energy balance of organisms and constitute various biochemical processes responsible for the formation, breakdown, and interconversion[14,15]. Further, 16 essential GO MF terms were included in Amino acid metabolism (e.g., "cysteine desulfurase activity", *UR* = 6.89) and RNA degradation (e.g., "3′-5′ exonuclease activity", *UR* = 5.27), which play an important role in energy balance through the reuse of RNA and amino acids. Purine (e.g., "ATP-dependent RNA helicase activity", *UR* = 5.04) and Pyrimidine (e.g., "thymidylate kinase activity", *UR* = 6.98) metabolisms are regarded as modular minimal cell model[16]. Generation of biological energy occurs mainly through the pathways contained in the Oxidative phosphorylation group[17]. These results demonstrate that a majority of these 160 essential GO MF terms are indispensable for the survival of an organism.

**Supplementary Text 5: Microarray expression data sets of 9 tumor types**

To identify genes with significant expression change between tumor and corresponding normal tissues, we collected 6 gene expression data sets, including 9 different tumor types, from GEO[18]. Each expression data set comprising ≥ 3 tumor samples and corresponding normal samples were obtained using the most comprehensive human expression array platform (HG U133 Plus 2.0; Supplementary Table S3). For each GeneChip array that passed quality control checks, quantile normalization was evaluated and gene expression values were calculated based on the $\log_2$ scale using the RMA algorithm[19]. Next, the Bioconductor method limma[20], based on a modified t-statistic, was utilized to measure the fold change value (FC) of each gene between tumor samples and corresponding normal samples. For each gene *g*, the FC is defined as $\mathrm{FC}_g = \mathrm{Average}(\log_2 T_g) - \mathrm{Average}(\log_2 N_g)$, where $T_g$ and $N_g$ is the expression in the *t* tumor samples ($T^1$, $T^2$,…,$T^t$) and *n* corresponding normal samples ($N^1$, $N^2$,…,$N^n$). The adjusted *P*-values were applied for multiple hypothesis testing using Benjamini and Hochberg's method[21] and the False Discovery Rate (FDR) was controlled at 5%. Finally, the genes with adjusted *P*-value <0.05 and fold change >1.3 are considered as significantly changed genes.

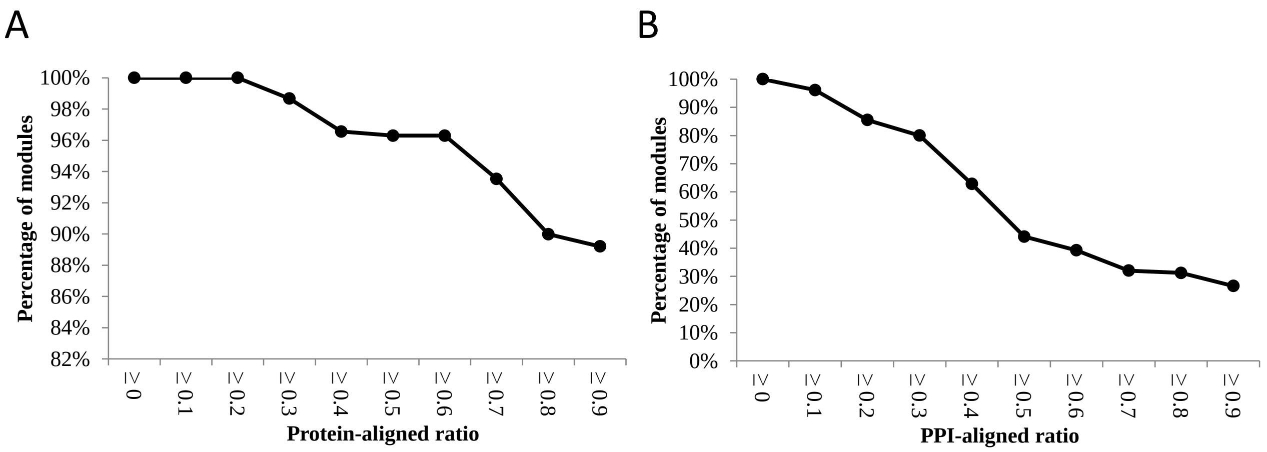


**Supplementary Figure S1. Evaluations of topological similarity.**

(A) The distribution of protein-aligned ratios and their fractions of 37,197 KEGG organism-specific structural complexes. (B) The distribution of PPI-aligned ratios and their fractions of 10,729 KEGG structural complexes, with intra-module interactions added using three PPI databases.

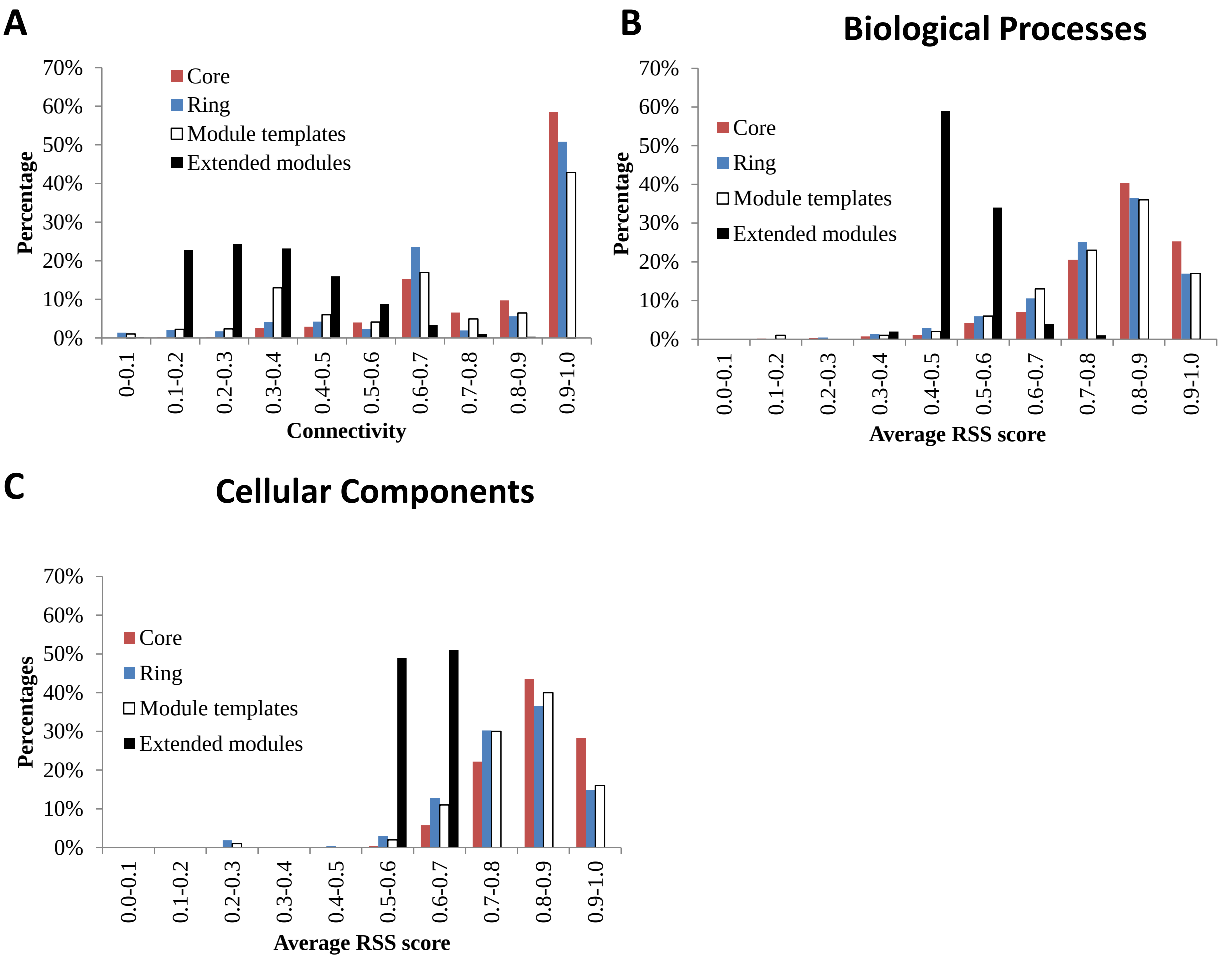


**Supplementary Figure S2. Distributions of connectivity ($C_t$) and average relative specificity similarity (AvgRSS) across core components and ring components of module templates and their extended modules.**

(A) $C_t$ distributions of core and ring components, module templates, and extended modules using 1,519 module templates; the extended module is a sub-network that includes one-layer extensions of PPIs and proteins of the module template. Distributions of AvgRSS scores of GO (B) biological processes and (C) cellular components for core and ring components, module templates, and extended modules.

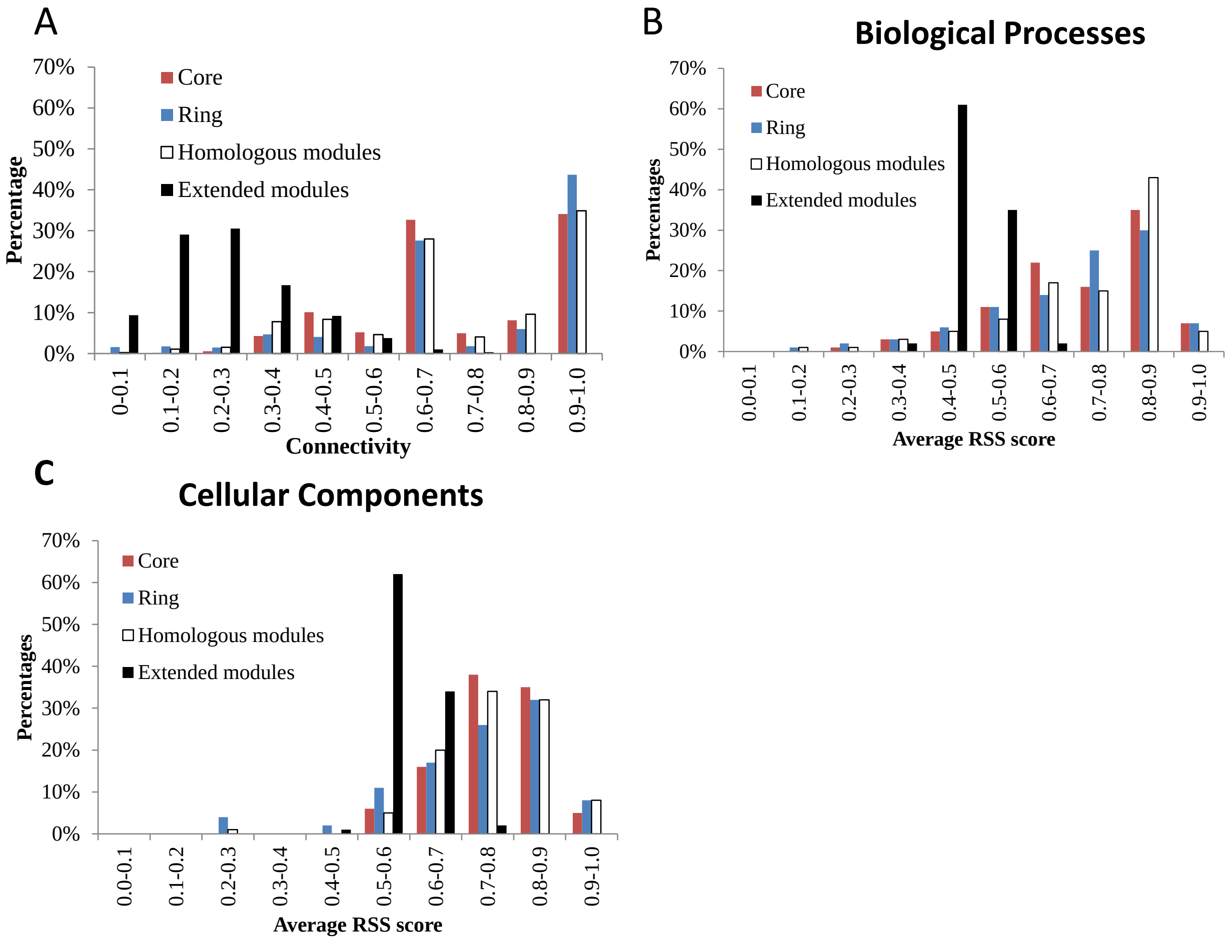


**Supplementary Figure S3. Connectivity ($C_t$) distributions and AvgRSS score distributions of GO Biological processes (BP) and Cellular Components (CCs) across core components and ring components of homologous modules and their extended modules.**

(A) $C_t$ distributions, (B) GO BP AvgRSS score distribution, and (C) GO CC AvgRSS score distributions of core components and ring components of 58,041 homologous modules and their respective extended modules.

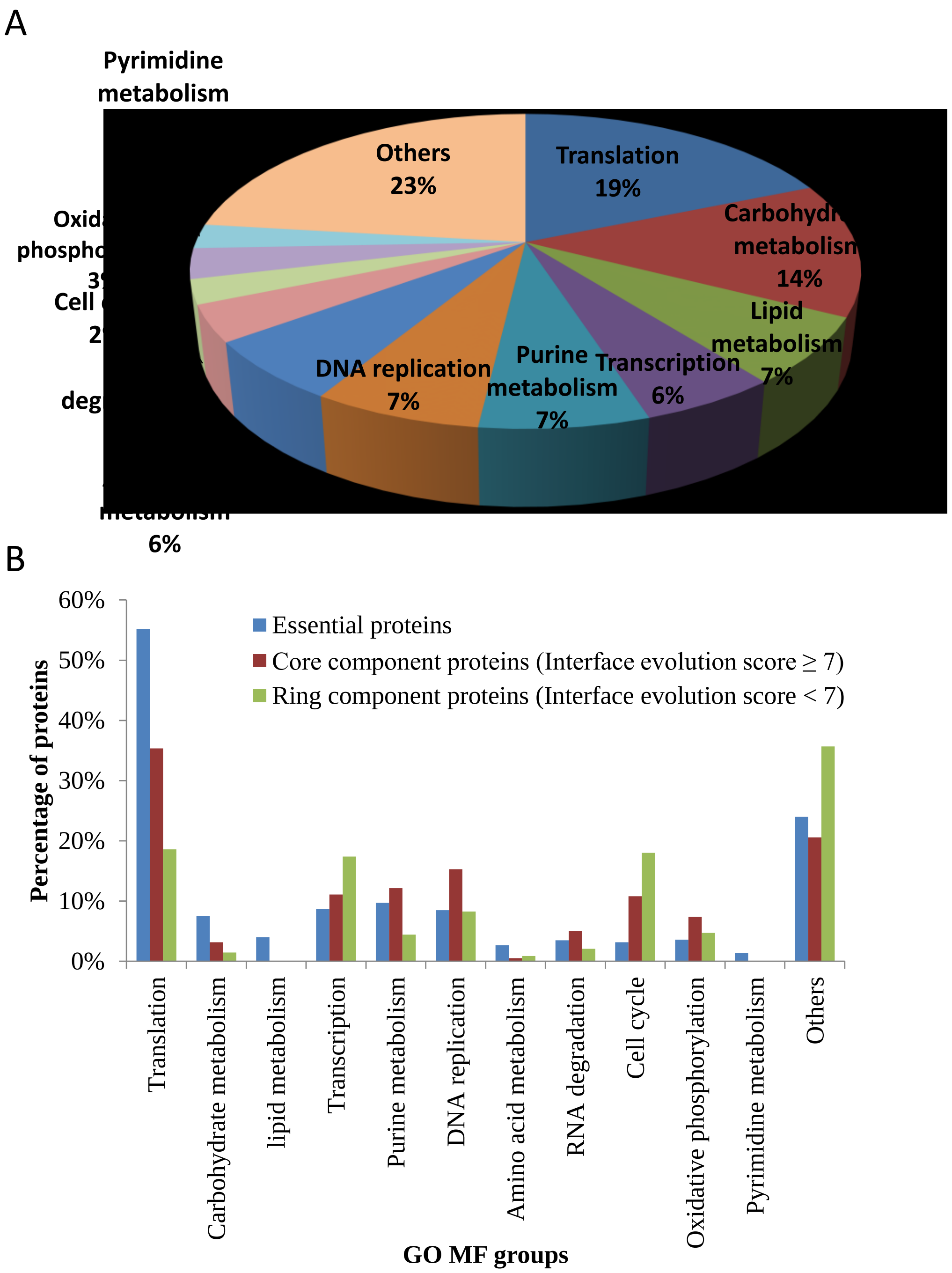


**Supplementary Figure S4. Gene ontology (GO) molecular function (MF) terms of essential proteins, core proteins, and ring proteins.**

(A) 12 groups of 160 essential GO MF terms taken from 8,364 essential proteins were sampled based on KEGG pathways and GO; (B) Percentages of GO MF groups in 3,366 essential proteins (blue), 379 core proteins (red), and 339 ring proteins (green).

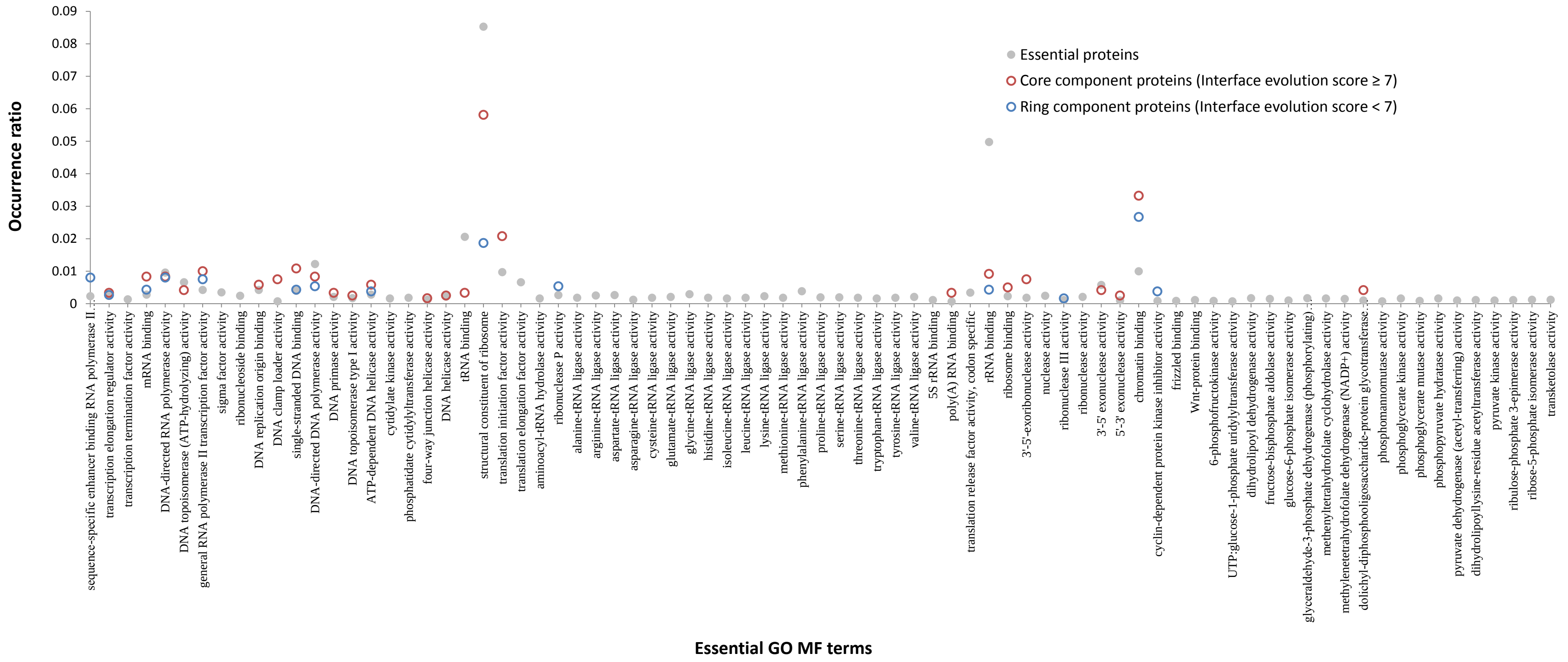


**Supplementary Figure S5. Occurrence ratios of 160 essential GO MF terms between essential proteins, core proteins, and ring proteins**

Occurrence ratios of each set are only labeled with the significant enrichment, as determined by p-values of $\leq 0.05$ (hypergeometric distribution) in each GO term.

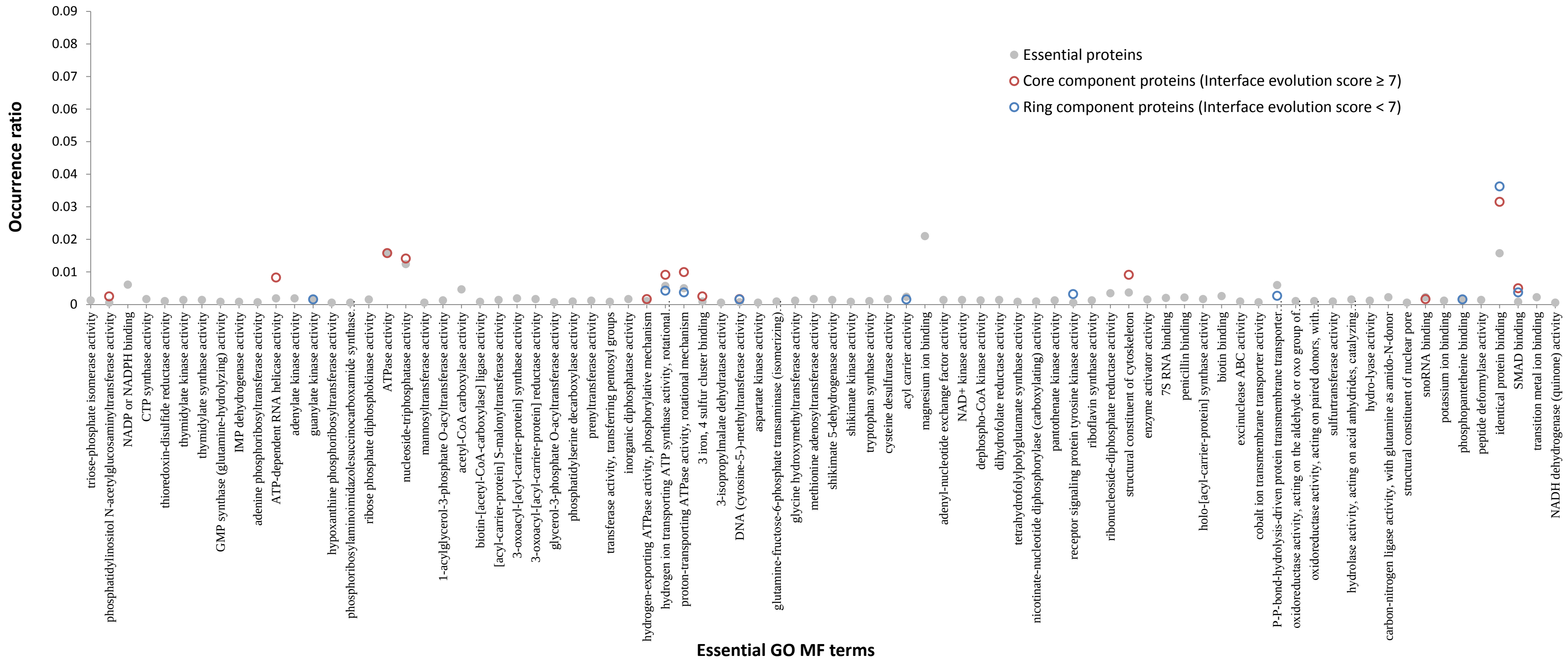


**Supplementary Figure S5. Occurrence ratios of 160 essential GO MF terms between essential proteins, core proteins, and ring proteins**

Occurrence ratios of each set are only labeled with the significant enrichment, as determined by p-values of ≤0.05 (hypergeometric distribution) in each GO term. (Continued)

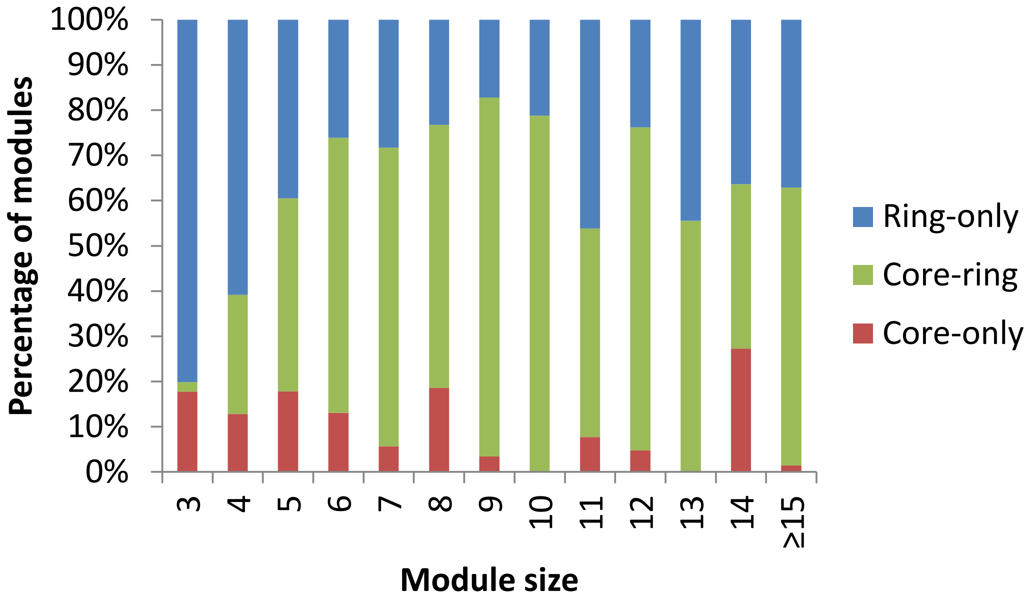


**Supplementary Figure S6. Module size distributions against percentages of modules with all proteins belonging to core proteins (core-only, red), ring proteins (ring-only, blue), or both (core–ring, green).**

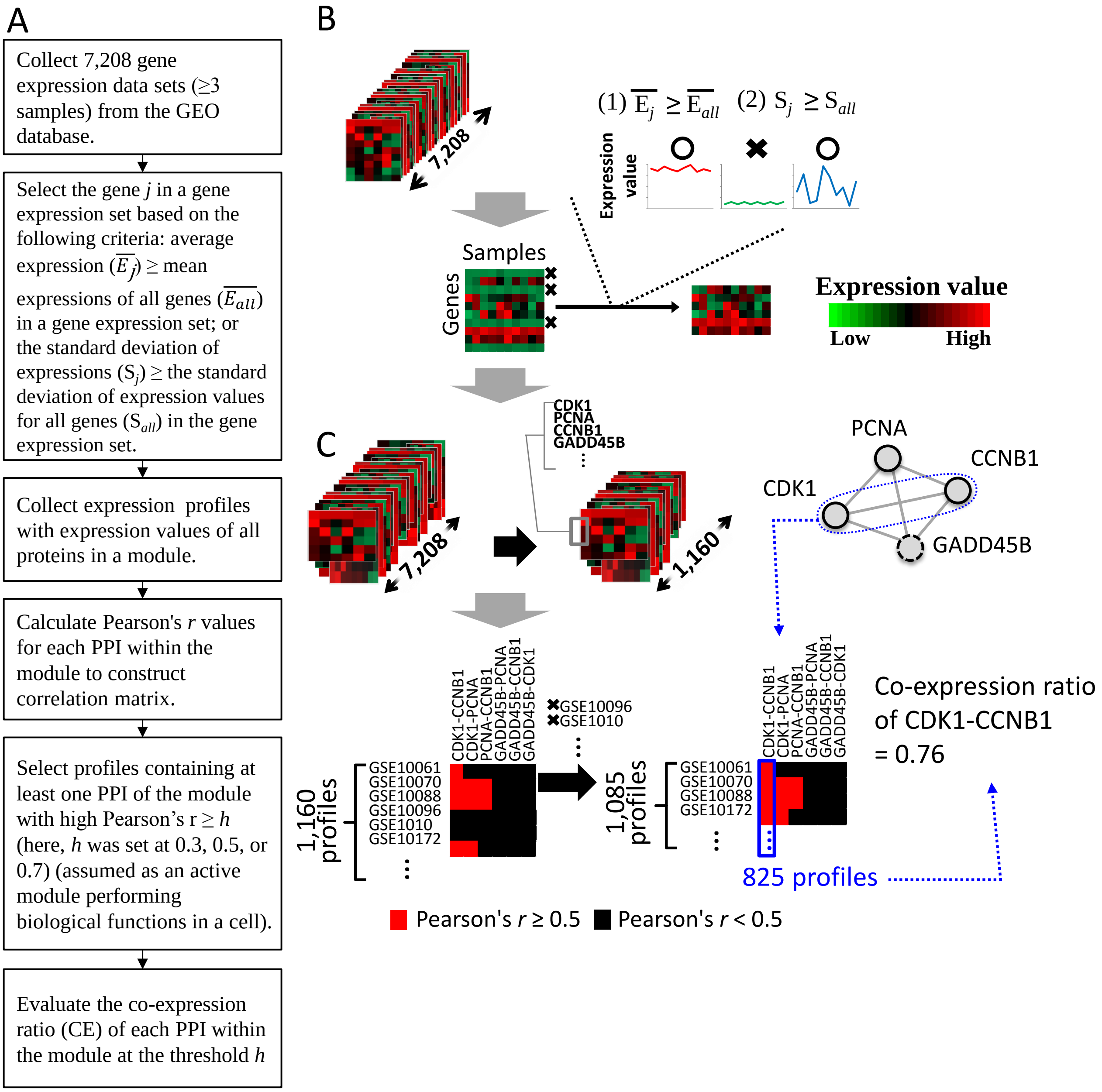


**Supplementary Figure S7. Gene co-expressions of core and ring PPIs in the modules using 7,208 *H. sapiens* gene sets from Gene Expression Omnibus (GEO).**

(A) The main procedure for collecting gene profiles and evaluating co-expression of core and ring PPIs in modules. (B) Gene expression profiles are collected by discarding non-significant genes with low expression and low expression variance. (C) Co-expression profiles of all protein pairs (PPIs) from the CDK1–PCNA–CCNB1–GADD45B module.

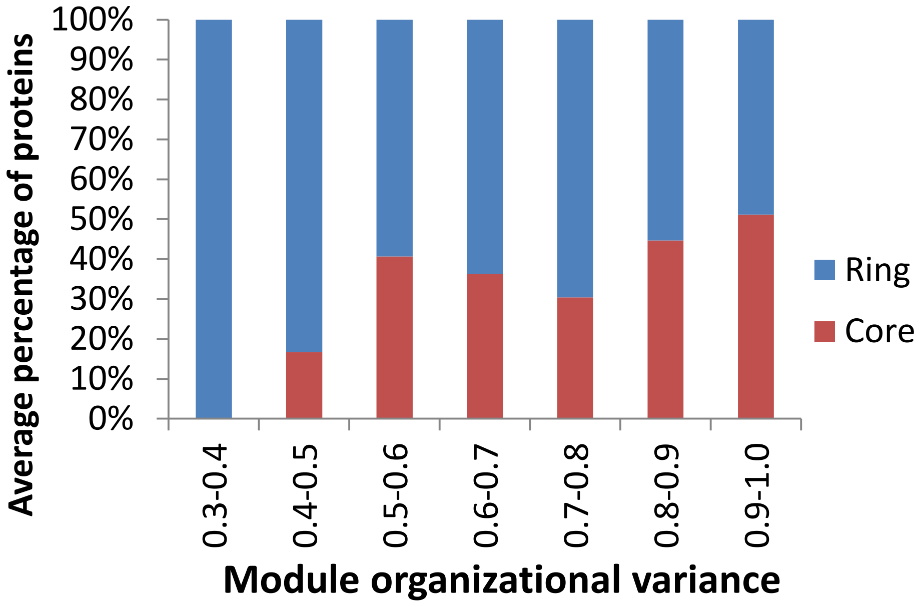


**Supplementary Figure S8. The relationship between module organizational variance and core/ring composition of modules.**

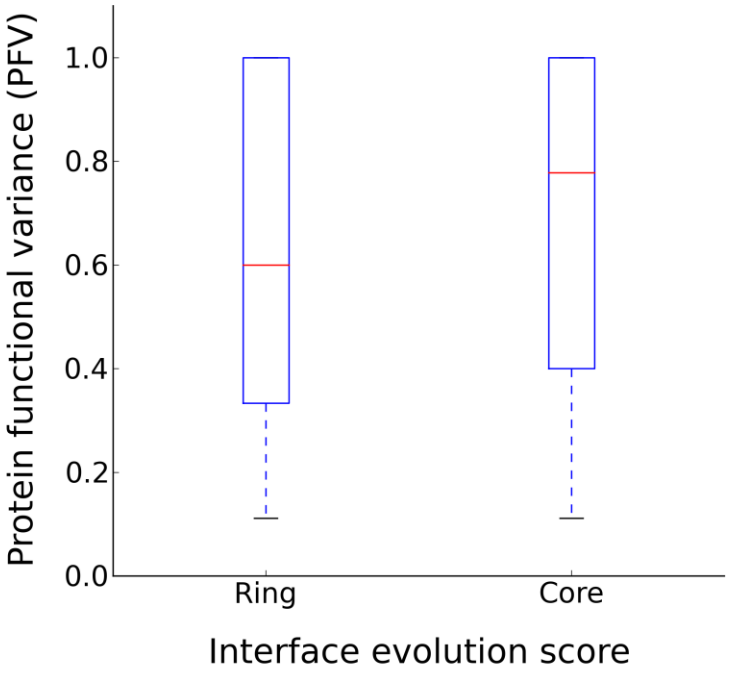


**Supplementary Figure S9. The boxplot of protein functional variance for ring and core proteins.**

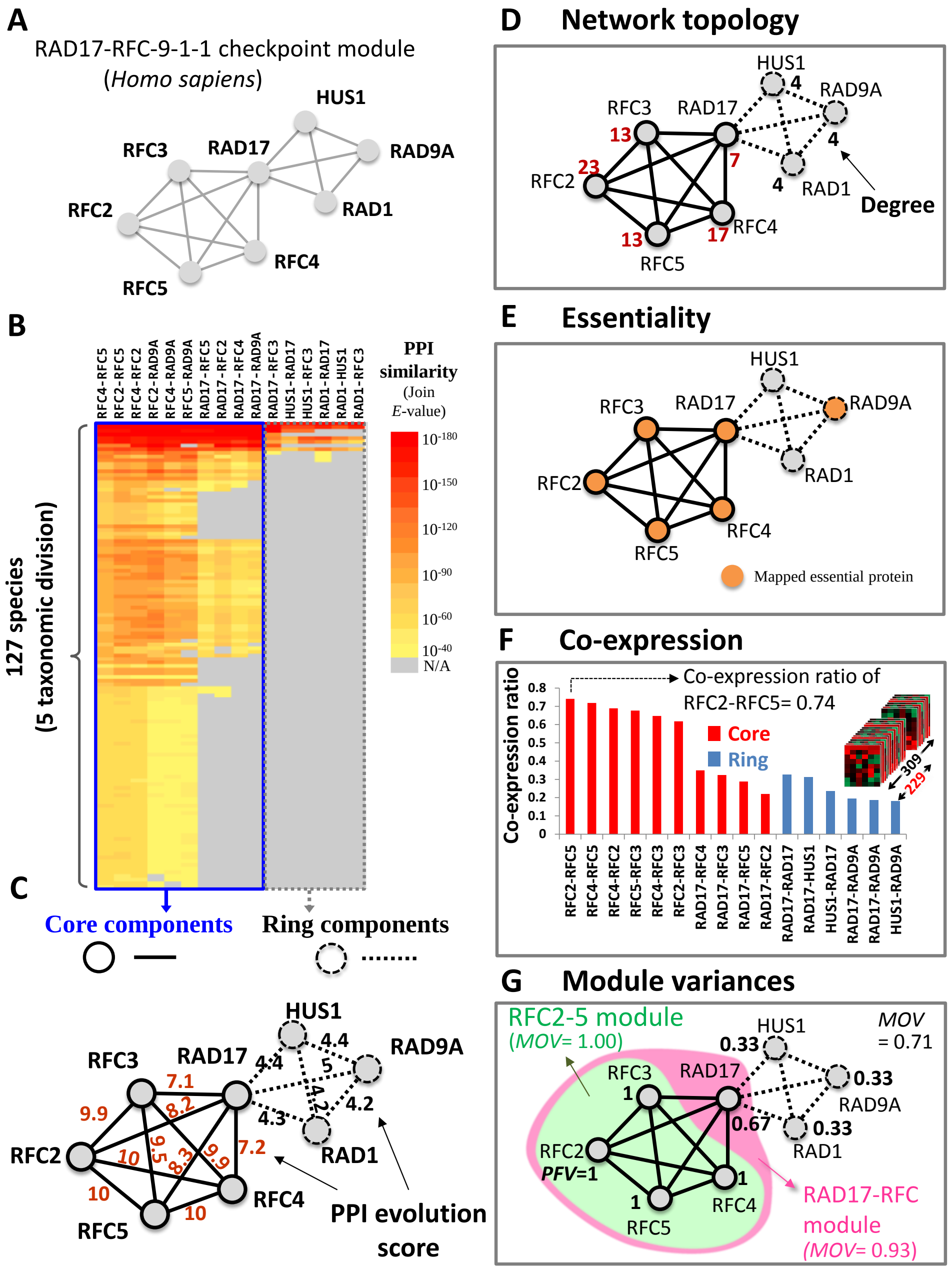


**Supplementary Figure S10. Characteristics and variance of core and ring components of RAD17–RFC-9-1-1 checkpoint module.**

(A) The RAD17–RFC-9-1-1 checkpoint module. (B) The module family profile includes 8 proteins and 16 PPI families. (C) Solid circles and lines denote the 5 core proteins and 10 core PPIs, respectively, and dashed circles and lines denote the 3 ring proteins and 6 ring PPIs, respectively. The PPI evolution scores are indicated. (D) Degrees of core and ring proteins in the human PPI network, including 2,391 proteins and 11,181 PPIs. (E) Orange circles indicate mapped essential proteins. (F) Co-expression ratios of 16 PPIs among 309 expression profiles selected from 7,208 gene sets. (G) The RAD17–RFC-9-1-1 supermodule comprises three modules with protein/module variance, including RFC2–5 (green), RAD17–RFC (pink), and RAD17–RFC-9-1-1. The solid circle and line denote the core protein and PPI, respectively. The dash circle and line indicate the ring protein and PPI, respectively.

**Supplementary Table S1. The 160 essential GO molecular function (MF) terms.**

| GO ID | GO term | Classification | Number of proteins (27 species genomes [a]) | Occurrence ratio [b] (27 species genomes; total 160,598 proteins) | Number of essential proteins | Occurrence ratio (essential proteins; total 8,364 proteins) | Unique ratio [c] (essential proteins) | Hypergeometric *P*-value (essential proteins) | Number of proteins in templates (*IES* ≥ 7) [d] | Occurrence ratio (*IES* ≥ 7; total 1,204 proteins) | Unique ratio (*IES* ≥ 7) |
|---|---|---|---|---|---|---|---|---|---|---|---|
| GO:0019843 | rRNA binding | Translation | 822 | 0.0051 | 416 | 0.0497 | 9.7173 | 5E-302 | 11 | 0.009129 | 1.783503 |
| GO:0004820 | glycine-tRNA ligase activity | Translation | 53 | 0.0003 | 24 | 0.0029 | 8.6948 | 3E-17 | 0 | 0 | 0 |
| GO:0000049 | tRNA binding | Translation | 394 | 0.0025 | 172 | 0.0206 | 8.3822 | 2E-111 | 4 | 0.00332 | 1.353059 |
| GO:0004818 | glutamate-tRNA ligase activity | Translation | 40 | 0.0002 | 17 | 0.0020 | 8.1605 | 4E-12 | 1 | 0.00083 | 3.331909 |
| GO:0004827 | proline-tRNA ligase activity | Translation | 38 | 0.0002 | 16 | 0.0019 | 8.0847 | 2E-11 | 1 | 0.00083 | 3.507272 |
| GO:0004832 | valine-tRNA ligase activity | Translation | 41 | 0.0003 | 17 | 0.0020 | 7.9614 | 7E-12 | 0 | 0 | 0 |
| GO:0004825 | methionine-tRNA ligase activity | Translation | 38 | 0.0002 | 15 | 0.0018 | 7.5794 | 3E-10 | 1 | 0.00083 | 3.507272 |
| GO:0004814 | arginine-tRNA ligase activity | Translation | 54 | 0.0003 | 21 | 0.0025 | 7.4671 | 1E-13 | 1 | 0.00083 | 2.468081 |
| GO:0004824 | lysine-tRNA ligase activity | Translation | 49 | 0.0003 | 19 | 0.0023 | 7.4453 | 2E-12 | 1 | 0.00083 | 2.719925 |
| GO:0004826 | phenylalanine-tRNA ligase activity | Translation | 88 | 0.0005 | 32 | 0.0038 | 6.9822 | 4E-19 | 0 | 0 | 0 |
| GO:0004823 | leucine-tRNA ligase activity | Translation | 43 | 0.0003 | 15 | 0.0018 | 6.6981 | 2E-9 | 1 | 0.00083 | 3.09945 |
| GO:0016149 | translation release factor activity, codon specific | Translation | 83 | 0.0005 | 28 | 0.0033 | 6.4775 | 7E-16 | 1 | 0.00083 | 1.605739 |
| GO:0004822 | isoleucine-tRNA ligase activity | Translation | 39 | 0.0002 | 13 | 0.0016 | 6.4004 | 5E-8 | 1 | 0.00083 | 3.417342 |
| GO:0004831 | tyrosine-tRNA ligase activity | Translation | 45 | 0.0003 | 15 | 0.0018 | 6.4004 | 4E-9 | 0 | 0 | 0 |
| GO:0004817 | cysteine-tRNA ligase activity | Translation | 47 | 0.0003 | 15 | 0.0018 | 6.1280 | 8E-9 | 0 | 0 | 0 |
| GO:0004526 | ribonuclease P activity | Translation | 71 | 0.0004 | 22 | 0.0026 | 5.9496 | 6E-12 | 0 | 0 | 0 |
| GO:0004829 | threonine-tRNA ligase activity | Translation | 49 | 0.0003 | 15 | 0.0018 | 5.8779 | 2E-8 | 0 | 0 | 0 |
| GO:0004816 | asparagine-tRNA ligase activity | Translation | 33 | 0.0002 | 10 | 0.0012 | 5.8185 | 4E-6 | 0 | 0 | 0 |

[a] The 27 species genome set (160,598 proteins annotated ≥ 1 GO MF terms) consists of 25 species in DEG and 2 species in module template set.
[b] The occurrence ratio of a GO MF term is defined as the number of proteins annotated this terms divided by the total number of proteins in the set.
[c] The unique ratio of a GO MF term is defined as the occurrence ratio of a GO MF term divided by the occurrence ratio in 27 species genome set.
[d] The proteins of module templates represent the core component proteins in module families with interface evolution score (*IES*) ≥ 7 and at least one GO MF term annotation in GO database.

**Supplementary Table S1.** **The 160 essential GO molecular function (MF) terms. (Continued)**

| GO ID | GO term | Classification | Number of proteins (27 species genomes [a]) | Occurrence ratio [b] (27 species genomes; total 160,598 proteins) | Number of essential proteins | Occurrence ratio (essential proteins; total 8,364 proteins) | Unique ratio [c] (essential proteins) | Hypergeometric *P*-value (essential proteins) | Number of proteins in templates (*IES* ≥ 7) [d] | Occurrence ratio (*IES* ≥ 7; total 1204 proteins) | Unique ratio (*IES* ≥ 7) |
|---|---|---|---|---|---|---|---|---|---|---|---|
| GO:0004828 | serine-tRNA ligase activity | Translation | 53 | 0.0003 | 16 | 0.0019 | 5.7966 | 7E-9 | 0 | 0 | 0 |
| GO:0004813 | alanine-tRNA ligase activity | Translation | 50 | 0.0003 | 15 | 0.0018 | 5.7603 | 2E-8 | 0 | 0 | 0 |
| GO:0004821 | histidine-tRNA ligase activity | Translation | 50 | 0.0003 | 15 | 0.0018 | 5.7603 | 2E-8 | 0 | 0 | 0 |
| GO:0004815 | aspartate-tRNA ligase activity | Translation | 77 | 0.0005 | 22 | 0.0026 | 5.4860 | 4E-11 | 1 | 0.00083 | 1.730862 |
| GO:0008097 | 5S rRNA binding | Translation | 34 | 0.0002 | 9 | 0.0011 | 5.0826 | 4E-5 | 1 | 0.00083 | 3.919893 |
| GO:0004830 | tryptophan-tRNA ligase activity | Translation | 50 | 0.0003 | 13 | 0.0016 | 4.9923 | 1E-6 | 0 | 0 | 0 |
| GO:0003735 | structural constituent of ribosome | Translation | 2903 | 0.0181 | 713 | 0.0852 | 4.7159 | 2E-274 | 70 | 0.058091 | 3.213691 |
| GO:0004045 | aminoacyl-tRNA hydrolase activity | Translation | 55 | 0.0003 | 13 | 0.0016 | 4.5384 | 4E-6 | 0 | 0 | 0 |
| GO:0008143 | poly(A) RNA binding | Translation | 31 | 0.0002 | 5 | 0.0006 | 3.0970 | 2E-2 | 4 | 0.00332 | 17.19695 |
| GO:0043022 | ribosome binding | Translation | 120 | 0.0007 | 19 | 0.0023 | 3.0402 | 1E-5 | 6 | 0.004979 | 6.663817 |
| GO:0003746 | translation elongation factor activity | Translation | 419 | 0.0026 | 55 | 0.0066 | 2.5204 | 4E-10 | 4 | 0.00332 | 1.272328 |
| GO:0003743 | translation initiation factor activity | Translation | 749 | 0.0047 | 81 | 0.0097 | 2.0765 | 7E-10 | 25 | 0.020747 | 4.448476 |
| GO:0004807 | triose-phosphate isomerase activity | Carbohydrate metabolism | 34 | 0.0002 | 11 | 0.0013 | 6.2121 | 7E-7 | 0 | 0 | 0 |
| GO:0004751 | ribose-5-phosphate isomerase activity | Carbohydrate metabolism | 31 | 0.0002 | 10 | 0.0012 | 6.1939 | 2E-6 | 0 | 0 | 0 |
| GO:0004148 | dihydrolipoyl dehydrogenase activity | Carbohydrate metabolism | 44 | 0.0003 | 14 | 0.0017 | 6.1094 | 3E-8 | 0 | 0 | 0 |
| GO:0004618 | phosphoglycerate kinase activity | Carbohydrate metabolism | 42 | 0.0003 | 13 | 0.0016 | 5.9432 | 1E-7 | 0 | 0 | 0 |
| GO:0004742 | dihydrolipoyllysine-residue acetyltransferase activity | Carbohydrate metabolism | 30 | 0.0002 | 9 | 0.0011 | 5.7603 | 1E-5 | 0 | 0 | 0 |
| GO:0004477 | methenyltetrahydrofolate cyclohydrolase activity | Carbohydrate metabolism | 45 | 0.0003 | 13 | 0.0016 | 5.5470 | 3E-7 | 0 | 0 | 0 |

[a] The 27 species genome set (160,598 proteins annotated ≥ 1 GO MF terms) consists of 25 species in DEG and 2 species in module template set.
[b] The occurrence ratio of a GO MF term is defined as the number of proteins annotated this terms divided by the total number of proteins in the set.
[c] The unique ratio of a GO MF term is defined as the occurrence ratio of a GO MF term divided by the occurrence ratio in 27 species genome set.
[d] The proteins of module templates represent the core component proteins in module families with interface evolution score (*IES*) ≥ 7 and at least one GO MF term annotation in GO database.

**Supplementary Table S1.** **The 160 essential GO molecular function (MF) terms. (Continued)**

| GO ID | GO term | Classification | Number of proteins (27 species genomes [a]) | Occurrence ratio [b] (27 species genomes; total 160,598 proteins) | Number of essential proteins | Occurrence ratio (essential proteins; total 8,364 proteins) | Unique ratio [c] (essential proteins) | Hypergeometric *P*-value (essential proteins) | Number of proteins in templates (*IES* ≥ 7) [d] | Occurrence ratio (*IES* ≥ 7; total 1,204 proteins) | Unique ratio (*IES* ≥ 7) |
|---|---|---|---|---|---|---|---|---|---|---|---|
| GO:0004488 | methylenetetrahydrofolate dehydrogenase (NADP+) activity | Carbohydrate metabolism | 42 | 0.0003 | 12 | 0.0014 | 5.4860 | 1E-6 | 0 | 0 | 0 |
| GO:0004802 | transketolase activity | Carbohydrate metabolism | 44 | 0.0003 | 10 | 0.0012 | 4.3639 | 7E-5 | 0 | 0 | 0 |
| GO:0004634 | phosphopyruvate hydratase activity | Carbohydrate metabolism | 58 | 0.0004 | 13 | 0.0016 | 4.3037 | 7E-6 | 0 | 0 | 0 |
| GO:0004347 | glucose-6-phosphate isomerase activity | Carbohydrate metabolism | 37 | 0.0002 | 8 | 0.0010 | 4.1516 | 5E-4 | 0 | 0 | 0 |
| GO:0003983 | UTP:glucose-1-phosphate uridylyltransferase activity | Carbohydrate metabolism | 32 | 0.0002 | 6 | 0.0007 | 3.6002 | 6E-3 | 0 | 0 | 0 |
| GO:0004615 | phosphomannomutase activity | Carbohydrate metabolism | 32 | 0.0002 | 6 | 0.0007 | 3.6002 | 6E-3 | 0 | 0 | 0 |
| GO:0004750 | ribulose-phosphate 3-epimerase activity | Carbohydrate metabolism | 52 | 0.0003 | 9 | 0.0011 | 3.3233 | 1E-3 | 0 | 0 | 0 |
| GO:0004365 | glyceraldehyde-3-phosphate dehydrogenase (phosphorylating) activity | Carbohydrate metabolism | 84 | 0.0005 | 14 | 0.0017 | 3.2002 | 1E-4 | 1 | 0.00083 | 1.586623 |
| GO:0017176 | phosphatidylinositol N-acetylglucosaminyltransferase activity | Carbohydrate metabolism | 33 | 0.0002 | 5 | 0.0006 | 2.9093 | 3E-2 | 3 | 0.00249 | 12.11603 |
| GO:0004739 | pyruvate dehydrogenase (acetyl-transferring) activity | Carbohydrate metabolism | 53 | 0.0003 | 8 | 0.0010 | 2.8983 | 6E-3 | 0 | 0 | 0 |
| GO:0004619 | phosphoglycerate mutase activity | Carbohydrate metabolism | 50 | 0.0003 | 7 | 0.0008 | 2.6882 | 1E-2 | 0 | 0 | 0 |
| GO:0004332 | fructose-bisphosphate aldolase activity | Carbohydrate metabolism | 86 | 0.0005 | 12 | 0.0014 | 2.6792 | 2E-3 | 0 | 0 | 0 |
| GO:0004579 | dolichyl-diphosphooligosaccharide-protein glycotransferase activity | Carbohydrate metabolism | 64 | 0.0004 | 8 | 0.0010 | 2.4001 | 2E-2 | 5 | 0.004149 | 10.41221 |
| GO:0003872 | 6-phosphofructokinase activity | Carbohydrate metabolism | 58 | 0.0004 | 7 | 0.0008 | 2.3174 | 3E-2 | 1 | 0.00083 | 2.297868 |
| GO:0050661 | NADP or NADPH binding | Carbohydrate metabolism | 449 | 0.0028 | 51 | 0.0061 | 2.1810 | 2E-7 | 2 | 0.00166 | 0.593659 |

[a] The 27 species genome set (160,598 proteins annotated ≥ 1 GO MF terms) consists of 25 species in DEG and 2 species in module template set.
[b] The occurrence ratio of a GO MF term is defined as the number of proteins annotated this terms divided by the total number of proteins in the set.
[c] The unique ratio of a GO MF term is defined as the occurrence ratio of a GO MF term divided by the occurrence ratio in 27 species genome set.
[d] The proteins of module templates represent the core component proteins in module families with interface evolution score (*IES*) ≥ 7 and at least one GO MF term annotation in GO database.

**Supplementary Table S1.** **The 160 essential GO molecular function (MF) terms. (Continued)**

| GO ID | GO term | Classification | Number of proteins (27 species genomes [a]) | Occurrence ratio [b] (27 species genomes; total 160,598 proteins) | Number of essential proteins | Occurrence ratio (essential proteins; total 8,364 proteins) | Unique ratio [c] (essential proteins) | Hypergeometric *P*-value (essential proteins) | Number of proteins in templates (*IES* ≥ 7) [d] | Occurrence ratio (*IES* ≥ 7; total 1,204 proteins) | Unique ratio (*IES* ≥ 7) |
|---|---|---|---|---|---|---|---|---|---|---|---|
| GO:0004743 | pyruvate kinase activity | Carbohydrate metabolism | 72 | 0.0004 | 8 | 0.0010 | 2.1335 | 3E-2 | 0 | 0 | 0 |
| GO:0003989 | acetyl-CoA carboxylase activity | lipid metabolism | 81 | 0.0005 | 39 | 0.0047 | 9.2450 | 2E-28 | 0 | 0 | 0 |
| GO:0004314 | [acyl-carrier-protein] S-malonyltransferase activity | lipid metabolism | 30 | 0.0002 | 12 | 0.0014 | 7.6804 | 1E-8 | 0 | 0 | 0 |
| GO:0004315 | 3-oxoacyl-[acyl-carrier-protein] synthase activity | lipid metabolism | 47 | 0.0003 | 16 | 0.0019 | 6.5365 | 9E-10 | 0 | 0 | 0 |
| GO:0004316 | 3-oxoacyl-[acyl-carrier-protein] reductase activity | lipid metabolism | 45 | 0.0003 | 14 | 0.0017 | 5.9737 | 4E-8 | 0 | 0 | 0 |
| GO:0004659 | prenyltransferase activity | lipid metabolism | 42 | 0.0003 | 10 | 0.0012 | 4.5717 | 5E-5 | 0 | 0 | 0 |
| GO:0004077 | biotin-[acetyl-CoA-carboxylase] ligase activity | lipid metabolism | 32 | 0.0002 | 7 | 0.0008 | 4.2002 | 1E-3 | 0 | 0 | 0 |
| GO:0004609 | phosphatidylserine decarboxylase activity | lipid metabolism | 41 | 0.0003 | 8 | 0.0010 | 3.7466 | 1E-3 | 0 | 0 | 0 |
| GO:0003841 | 1-acylglycerol-3-phosphate O-acyltransferase activity | lipid metabolism | 58 | 0.0004 | 11 | 0.0013 | 3.6416 | 2E-4 | 0 | 0 | 0 |
| GO:0004366 | glycerol-3-phosphate O-acyltransferase activity | lipid metabolism | 41 | 0.0003 | 6 | 0.0007 | 2.8099 | 2E-2 | 0 | 0 | 0 |
| GO:0000030 | mannosyltransferase activity | lipid metabolism | 37 | 0.0002 | 5 | 0.0006 | 2.5947 | 4E-2 | 0 | 0 | 0 |
| GO:0016763 | transferase activity, transferring pentosyl groups | lipid metabolism | 56 | 0.0003 | 7 | 0.0008 | 2.4001 | 3E-2 | 0 | 0 | 0 |
| GO:0003918 | DNA topoisomerase (ATP-hydrolyzing) activity | Transcription | 122 | 0.0008 | 55 | 0.0066 | 8.6562 | 1E-37 | 5 | 0.004149 | 5.462145 |

[a] The 27 species genome set (160,598 proteins annotated ≥ 1 GO MF terms) consists of 25 species in DEG and 2 species in module template set.
[b] The occurrence ratio of a GO MF term is defined as the number of proteins annotated this terms divided by the total number of proteins in the set.
[c] The unique ratio of a GO MF term is defined as the occurrence ratio of a GO MF term divided by the occurrence ratio in 27 species genome set.
[d] The proteins of module templates represent the core component proteins in module families with interface evolution score (*IES*) ≥ 7 and at least one GO MF term annotation in GO database.

**Supplementary Table S1.** **The 160 essential GO molecular function (MF) terms. (Continued)**

| GO ID | GO term | Classification | Number of proteins (27 species genomes [a]) | Occurrence ratio [b] (27 species genomes; total 160,598 proteins) | Number of essential proteins | Occurrence ratio (essential proteins; total 8,364 proteins) | Unique ratio [c] (essential proteins) | Hypergeometric *P*-value (essential proteins) | Number of proteins in templates (*IES* ≥ 7) [d] | Occurrence ratio (*IES* ≥ 7; total 1,204 proteins) | Unique ratio (*IES* ≥ 7) |
|---|---|---|---|---|---|---|---|---|---|---|---|
| GO:0016251 | general RNA polymerase II transcription factor activity | Transcription | 103 | 0.0006 | 35 | 0.0042 | 6.5246 | 1E-19 | 12 | 0.009959 | 15.52734 |
| GO:0003715 | transcription termination factor activity | Transcription | 37 | 0.0002 | 11 | 0.0013 | 5.7084 | 2E-6 | 0 | 0 | 0 |
| GO:0032549 | ribonucleoside binding | Transcription | 69 | 0.0004 | 20 | 0.0024 | 5.5655 | 2E-10 | 1 | 0.00083 | 1.931541 |
| GO:0016987 | sigma factor activity | Transcription | 136 | 0.0008 | 29 | 0.0035 | 4.0944 | 8E-11 | 0 | 0 | 0 |
| GO:0003711 | transcription elongation regulator activity | Transcription | 105 | 0.0007 | 20 | 0.0024 | 3.6574 | 5E-7 | 4 | 0.00332 | 5.077194 |
| GO:0003899 | DNA-directed RNA polymerase activity | Transcription | 473 | 0.0029 | 80 | 0.0096 | 3.2475 | 2E-20 | 10 | 0.008299 | 2.817682 |
| GO:0003729 | mRNA binding | Transcription | 162 | 0.0010 | 23 | 0.0027 | 2.7261 | 1E-5 | 10 | 0.008299 | 8.226935 |
| GO:0003705 | sequence-specific enhancer binding RNA polymerase II transcription factor activity | Transcription | 151 | 0.0009 | 19 | 0.0023 | 2.4160 | 3E-4 | 0 | 0 | 0 |
| GO:0004004 | ATP-dependent RNA helicase activity | Purine metabolism | 61 | 0.0004 | 16 | 0.0019 | 5.0364 | 6E-8 | 10 | 0.008299 | 21.84858 |
| GO:0003922 | GMP synthase (glutamine-hydrolyzing) activity | Purine metabolism | 30 | 0.0002 | 7 | 0.0008 | 4.4803 | 7E-4 | 0 | 0 | 0 |
| GO:0004385 | guanylate kinase activity | Purine metabolism | 63 | 0.0004 | 14 | 0.0017 | 4.2669 | 4E-6 | 0 | 0 | 0 |
| GO:0003999 | adenine phosphoribosyltransferase activity | Purine metabolism | 33 | 0.0002 | 6 | 0.0007 | 3.4911 | 7E-3 | 0 | 0 | 0 |
| GO:0004017 | adenylate kinase activity | Purine metabolism | 89 | 0.0006 | 16 | 0.0019 | 3.4519 | 1E-5 | 0 | 0 | 0 |
| GO:0004639 | phosphoribosylaminoimidazolesuccinocarboxamide synthase activity | Purine metabolism | 30 | 0.0002 | 5 | 0.0006 | 3.2002 | 2E-2 | 0 | 0 | 0 |
| GO:0004749 | ribose phosphate diphosphokinase activity | Purine metabolism | 78 | 0.0005 | 13 | 0.0016 | 3.2002 | 2E-4 | 0 | 0 | 0 |
| GO:0003938 | IMP dehydrogenase activity | Purine metabolism | 46 | 0.0003 | 7 | 0.0008 | 2.9219 | 9E-3 | 0 | 0 | 0 |

[a] The 27 species genome set (160,598 proteins annotated ≥ 1 GO MF terms) consists of 25 species in DEG and 2 species in module template set.
[b] The occurrence ratio of a GO MF term is defined as the number of proteins annotated this terms divided by the total number of proteins in the set.
[c] The unique ratio of a GO MF term is defined as the occurrence ratio of a GO MF term divided by the occurrence ratio in 27 species genome set.
[d] The proteins of module templates represent the core component proteins in module families with interface evolution score (*IES*) ≥ 7 and at least one GO MF term annotation in GO database.

**Supplementary Table S1. The 160 essential GO molecular function (MF) terms. (Continued)**

| GO ID | GO term | Classification | Number of proteins (27 species genomes [a]) | Occurrence ratio [b] (27 species genomes; total 160,598 proteins) | Number of essential proteins | Occurrence ratio (essential proteins; total 8,364 proteins) | Unique ratio [c] (essential proteins) | Hypergeometric *P*-value (essential proteins) | Number of proteins in templates (*IES* ≥ 7) [d] | Occurrence ratio (*IES* ≥ 7; total 1,204 proteins) | Unique ratio (*IES* ≥ 7) |
|---|---|---|---|---|---|---|---|---|---|---|---|
| GO:0004422 | hypoxanthine phosphoribosyltransferase activity | Purine metabolism | 33 | 0.0002 | 5 | 0.0006 | 2.9093 | 3E-2 | 0 | 0 | 0 |
| GO:0016887 | ATPase activity | Purine metabolism | 1174 | 0.0073 | 134 | 0.0160 | 2.1916 | 4E-17 | 19 | 0.015768 | 2.156943 |
| GO:0017111 | nucleoside-triphosphatase activity | Purine metabolism | 952 | 0.0059 | 104 | 0.0124 | 2.0976 | 2E-12 | 17 | 0.014108 | 2.379935 |
| GO:0003688 | DNA replication origin binding | DNA replication | 58 | 0.0004 | 35 | 0.0042 | 11.5869 | 3E-30 | 7 | 0.005809 | 16.08508 |
| GO:0004127 | cytidylate kinase activity | DNA replication | 30 | 0.0002 | 13 | 0.0016 | 8.3205 | 1E-9 | 0 | 0 | 0 |
| GO:0004605 | phosphatidate cytidylyltransferase activity | DNA replication | 46 | 0.0003 | 15 | 0.0018 | 6.2612 | 6E-9 | 0 | 0 | 0 |
| GO:0003896 | DNA primase activity | DNA replication | 59 | 0.0004 | 17 | 0.0020 | 5.5325 | 5E-9 | 4 | 0.00332 | 9.035685 |
| GO:0009378 | four-way junction helicase activity | DNA replication | 45 | 0.0003 | 10 | 0.0012 | 4.2669 | 9E-5 | 2 | 0.00166 | 5.923393 |
| GO:0003678 | DNA helicase activity | DNA replication | 89 | 0.0006 | 18 | 0.0022 | 3.8834 | 7E-7 | 3 | 0.00249 | 4.492461 |
| GO:0003887 | DNA-directed DNA polymerase activity | DNA replication | 518 | 0.0032 | 102 | 0.0122 | 3.7809 | 4E-31 | 10 | 0.008299 | 2.572902 |
| GO:0003917 | DNA topoisomerase type I activity | DNA replication | 83 | 0.0005 | 14 | 0.0017 | 3.2387 | 9E-5 | 3 | 0.00249 | 4.817217 |
| GO:0003689 | DNA clamp loader activity | DNA replication | 47 | 0.0003 | 6 | 0.0007 | 2.4512 | 3E-2 | 9 | 0.007469 | 25.521 |
| GO:0003697 | single-stranded DNA binding | DNA replication | 271 | 0.0017 | 33 | 0.0039 | 2.3381 | 6E-6 | 13 | 0.010788 | 6.39333 |
| GO:0004003 | ATP-dependent DNA helicase activity | DNA replication | 219 | 0.0014 | 23 | 0.0027 | 2.0166 | 1E-3 | 7 | 0.005809 | 4.259975 |
| GO:0031071 | cysteine desulfurase activity | Amino acid metabolism | 39 | 0.0002 | 14 | 0.0017 | 6.8927 | 5E-9 | 0 | 0 | 0 |
| GO:0004478 | methionine adenosyltransferase activity | Amino acid metabolism | 51 | 0.0003 | 14 | 0.0017 | 5.2709 | 2E-7 | 0 | 0 | 0 |

[a] The 27 species genome set (160,598 proteins annotated ≥ 1 GO MF terms) consists of 25 species in DEG and 2 species in module template set.
[b] The occurrence ratio of a GO MF term is defined as the number of proteins annotated this terms divided by the total number of proteins in the set.
[c] The unique ratio of a GO MF term is defined as the occurrence ratio of a GO MF term divided by the occurrence ratio in 27 species genome set.
[d] The proteins of module templates represent the core component proteins in module families with interface evolution score (*IES*) ≥ 7 and at least one GO MF term annotation in GO database.

**Supplementary Table S1.** **The 160 essential GO molecular function (MF) terms. (Continued)**

| GO ID | GO term | Classification | Number of proteins (27 species genomes [a]) | Occurrence ratio [b] (27 species genomes; total 160,598 proteins) | Number of essential proteins | Occurrence ratio (essential proteins; total 8,364 proteins) | Unique ratio [c] (essential proteins) | Hypergeometric *P*-value (essential proteins) | Number of proteins in templates (*IES* ≥ 7) [d] | Occurrence ratio (*IES* ≥ 7; total 1,204 proteins) | Unique ratio (*IES* ≥ 7) |
|---|---|---|---|---|---|---|---|---|---|---|---|
| GO:0004764 | shikimate 5-dehydrogenase activity | Amino acid metabolism | 45 | 0.0003 | 12 | 0.0014 | 5.1203 | 2E-6 | 0 | 0 | 0 |
| GO:0004834 | tryptophan synthase activity | Amino acid metabolism | 39 | 0.0002 | 9 | 0.0011 | 4.4310 | 1E-4 | 0 | 0 | 0 |
| GO:0004360 | glutamine-fructose-6-phosphate transaminase (isomerizing) activity | Amino acid metabolism | 35 | 0.0002 | 8 | 0.0010 | 4.3888 | 4E-4 | 0 | 0 | 0 |
| GO:0003886 | DNA (cytosine-5-)-methyltransferase activity | Amino acid metabolism | 36 | 0.0002 | 6 | 0.0007 | 3.2002 | 1E-2 | 2 | 0.00166 | 7.404242 |
| GO:0004372 | glycine hydroxymethyltransferase activity | Amino acid metabolism | 61 | 0.0004 | 10 | 0.0012 | 3.1477 | 1E-3 | 0 | 0 | 0 |
| GO:0003861 | 3-isopropylmalate dehydratase activity | Amino acid metabolism | 34 | 0.0002 | 5 | 0.0006 | 2.8237 | 3E-2 | 0 | 0 | 0 |
| GO:0004072 | aspartate kinase activity | Amino acid metabolism | 37 | 0.0002 | 5 | 0.0006 | 2.5947 | 4E-2 | 0 | 0 | 0 |
| GO:0004765 | shikimate kinase activity | Amino acid metabolism | 52 | 0.0003 | 7 | 0.0008 | 2.5848 | 2E-2 | 0 | 0 | 0 |
| GO:0008408 | 3'-5' exonuclease activity | RNA degradation | 175 | 0.0011 | 48 | 0.0057 | 5.2666 | 9E-22 | 5 | 0.004149 | 3.807896 |
| GO:0000175 | 3'-5'-exoribonuclease activity | RNA degradation | 108 | 0.0007 | 15 | 0.0018 | 2.6668 | 5E-4 | 9 | 0.007469 | 11.10636 |
| GO:0004540 | ribonuclease activity | RNA degradation | 128 | 0.0008 | 17 | 0.0020 | 2.5501 | 4E-4 | 0 | 0 | 0 |
| GO:0004525 | ribonuclease III activity | RNA degradation | 70 | 0.0004 | 9 | 0.0011 | 2.4687 | 1E-2 | 1 | 0.00083 | 1.903948 |
| GO:0008409 | 5'-3' exonuclease activity | RNA degradation | 67 | 0.0004 | 8 | 0.0010 | 2.2927 | 2E-2 | 3 | 0.00249 | 5.967598 |
| GO:0004518 | nuclease activity | RNA degradation | 177 | 0.0011 | 20 | 0.0024 | 2.1696 | 1E-3 | 1 | 0.00083 | 0.752974 |
| GO:0005109 | frizzled binding | Cell cycle | 40 | 0.0002 | 7 | 0.0008 | 3.3602 | 4E-3 | 0 | 0 | 0 |

[a] The 27 species genome set (160,598 proteins annotated ≥ 1 GO MF terms) consists of 25 species in DEG and 2 species in module template set.
[b] The occurrence ratio of a GO MF term is defined as the number of proteins annotated this terms divided by the total number of proteins in the set.
[c] The unique ratio of a GO MF term is defined as the occurrence ratio of a GO MF term divided by the occurrence ratio in 27 species genome set.
[d] The proteins of module templates represent the core component proteins in module families with interface evolution score (*IES*) ≥ 7 and at least one GO MF term annotation in GO database.

**Supplementary Table S1.** **The 160 essential GO molecular function (MF) terms. (Continued)**

| GO ID | GO term | Classification | Number of proteins (27 species genomes [a]) | Occurrence ratio [b] (27 species genomes; total 160,598 proteins) | Number of essential proteins | Occurrence ratio (essential proteins; total 8,364 proteins) | Unique ratio [c] (essential proteins) | Hypergeometric *P*-value (essential proteins) | Number of proteins in templates (*IES* ≥ 7) [d] | Occurrence ratio (*IES* ≥ 7; total 1,204 proteins) | Unique ratio (*IES* ≥ 7) |
|---|---|---|---|---|---|---|---|---|---|---|---|
| GO:0004861 | cyclin-dependent protein kinase inhibitor activity | Cell cycle | 50 | 0.0003 | 7 | 0.0008 | 2.6882 | 1E-2 | 1 | 0.00083 | 2.665527 |
| GO:0017147 | Wnt-protein binding | Cell cycle | 70 | 0.0004 | 9 | 0.0011 | 2.4687 | 1E-2 | 0 | 0 | 0 |
| GO:0003682 | chromatin binding | Cell cycle | 710 | 0.0044 | 83 | 0.0099 | 2.2446 | 1E-11 | 40 | 0.033195 | 7.508527 |
| GO:0004427 | inorganic diphosphatase activity | Oxidative phosphorylation | 65 | 0.0004 | 14 | 0.0017 | 4.1356 | 5E-6 | 0 | 0 | 0 |
| GO:0051538 | 3 iron, 4 sulfur cluster binding | Oxidative phosphorylation | 43 | 0.0003 | 8 | 0.0010 | 3.5723 | 2E-3 | 3 | 0.00249 | 9.29835 |
| GO:0046933 | hydrogen ion transporting ATP synthase activity, rotational mechanism | Oxidative phosphorylation | 280 | 0.0017 | 48 | 0.0057 | 3.2916 | 4E-13 | 11 | 0.009129 | 5.235857 |
| GO:0046961 | proton-transporting ATPase activity, rotational mechanism | Oxidative phosphorylation | 285 | 0.0018 | 42 | 0.0050 | 2.8296 | 1E-9 | 12 | 0.009959 | 5.611636 |
| GO:0008553 | hydrogen-exporting ATPase activity, phosphorylative mechanism | Oxidative phosphorylation | 86 | 0.0005 | 9 | 0.0011 | 2.0094 | 4E-2 | 2 | 0.00166 | 3.09945 |
| GO:0004798 | thymidylate kinase activity | Pyrimidine metabolism | 33 | 0.0002 | 12 | 0.0014 | 6.9822 | 5E-8 | 0 | 0 | 0 |
| GO:0004799 | thymidylate synthase activity | Pyrimidine metabolism | 33 | 0.0002 | 12 | 0.0014 | 6.9822 | 5E-8 | 0 | 0 | 0 |
| GO:0003883 | CTP synthase activity | Pyrimidine metabolism | 40 | 0.0002 | 14 | 0.0017 | 6.7204 | 7E-9 | 0 | 0 | 0 |
| GO:0004791 | thioredoxin-disulfide reductase activity | Pyrimidine metabolism | 49 | 0.0003 | 9 | 0.0011 | 3.5267 | 9E-4 | 0 | 0 | 0 |
| GO:0030515 | snoRNA binding | Others | 49 | 0.0003 | 19 | 0.0023 | 7.4453 | 2E-12 | 2 | 0.00166 | 5.439851 |
| GO:0042586 | peptide deformylase activity | Others | 31 | 0.0002 | 12 | 0.0014 | 7.4327 | 2E-8 | 0 | 0 | 0 |
| GO:0004146 | dihydrofolate reductase activity | Others | 34 | 0.0002 | 12 | 0.0014 | 6.7769 | 7E-8 | 0 | 0 | 0 |
| GO:0004746 | riboflavin synthase activity | Others | 32 | 0.0002 | 11 | 0.0013 | 6.6004 | 4E-7 | 0 | 0 | 0 |
| GO:0004748 | ribonucleoside-diphosphate reductase activity | Others | 89 | 0.0006 | 29 | 0.0035 | 6.2565 | 6E-16 | 0 | 0 | 0 |

[a] The 27 species genome set (160,598 proteins annotated ≥ 1 GO MF terms) consists of 25 species in DEG and 2 species in module template set.
[b] The occurrence ratio of a GO MF term is defined as the number of proteins annotated this terms divided by the total number of proteins in the set.
[c] The unique ratio of a GO MF term is defined as the occurrence ratio of a GO MF term divided by the occurrence ratio in 27 species genome set.
[d] The proteins of module templates represent the core component proteins in module families with interface evolution score (*IES*) ≥ 7 and at least one GO MF term annotation in GO database.

**Supplementary Table S1.** **The 160 essential GO molecular function (MF) terms. (Continued)**

| GO ID | GO term | Classification | Number of proteins (27 species genomes [a]) | Occurrence ratio [b] (27 species genomes; total 160,598 proteins) | Number of essential proteins | Occurrence ratio (essential proteins; total 8,364 proteins) | Unique ratio [c] (essential proteins) | Hypergeometric *P*-value (essential proteins) | Number of proteins in templates (*IES* ≥ 7) [d] | Occurrence ratio (*IES* ≥ 7; total 1,204 proteins) | Unique ratio (*IES* ≥ 7) |
|---|---|---|---|---|---|---|---|---|---|---|---|
| GO:0004140 | dephospho-CoA kinase activity | Others | 35 | 0.0002 | 11 | 0.0013 | 6.0346 | 1E-6 | 0 | 0 | 0 |
| GO:0008897 | holo-[acyl-carrier-protein] synthase activity | Others | 47 | 0.0003 | 14 | 0.0017 | 5.7195 | 7E-8 | 0 | 0 | 0 |
| GO:0000774 | adenyl-nucleotide exchange factor activity | Others | 42 | 0.0003 | 12 | 0.0014 | 5.4860 | 1E-6 | 0 | 0 | 0 |
| GO:0015450 | P-P-bond-hydrolysis-driven protein transmembrane transporter activity | Others | 189 | 0.0012 | 50 | 0.0060 | 5.0797 | 7E-22 | 2 | 0.00166 | 1.410332 |
| GO:0016836 | hydro-lyase activity | Others | 40 | 0.0002 | 10 | 0.0012 | 4.8003 | 3E-5 | 0 | 0 | 0 |
| GO:0016783 | sulfurtransferase activity | Others | 33 | 0.0002 | 8 | 0.0010 | 4.6548 | 2E-4 | 0 | 0 | 0 |
| GO:0004594 | pantothenate kinase activity | Others | 48 | 0.0003 | 11 | 0.0013 | 4.4003 | 3E-5 | 0 | 0 | 0 |
| GO:0016624 | oxidoreductase activity, acting on the aldehyde or oxo group of donors, disulfide as acceptor | Others | 40 | 0.0002 | 9 | 0.0011 | 4.3202 | 2E-4 | 0 | 0 | 0 |
| GO:0008312 | 7S RNA binding | Others | 76 | 0.0005 | 17 | 0.0020 | 4.2950 | 3E-7 | 1 | 0.00083 | 1.753636 |
| GO:0008658 | penicillin binding | Others | 86 | 0.0005 | 18 | 0.0022 | 4.0188 | 4E-7 | 0 | 0 | 0 |
| GO:0009374 | biotin binding | Others | 109 | 0.0007 | 22 | 0.0026 | 3.8755 | 4E-8 | 0 | 0 | 0 |
| GO:0016709 | oxidoreductase activity, acting on paired donors, with incorporation or reduction of molecular oxygen, NADH or NADPH as one donor, and incorporation of one atom of oxygen | Others | 50 | 0.0003 | 9 | 0.0011 | 3.4562 | 1E-3 | 0 | 0 | 0 |
| GO:0046914 | transition metal ion binding | Others | 106 | 0.0007 | 19 | 0.0023 | 3.4417 | 2E-6 | 0 | 0 | 0 |
| GO:0003951 | NAD+ kinase activity | Others | 67 | 0.0004 | 12 | 0.0014 | 3.4390 | 2E-4 | 0 | 0 | 0 |
| GO:0016820 | hydrolase activity, acting on acid anhydrides, catalyzing transmembrane movement of substances | Others | 74 | 0.0005 | 13 | 0.0016 | 3.3732 | 1E-4 | 0 | 0 | 0 |
| GO:0051087 | chaperone binding | Others | 137 | 0.0009 | 24 | 0.0029 | 3.3637 | 2E-7 | 5 | 0.004149 | 4.8641 |
| GO:0016884 | carbon-nitrogen ligase activity, with glutamine as amido-N-donor | Others | 109 | 0.0007 | 19 | 0.0023 | 3.3470 | 4E-6 | 0 | 0 | 0 |

[a] The 27 species genome set (160,598 proteins annotated ≥ 1 GO MF terms) consists of 25 species in DEG and 2 species in module template set.
[b] The occurrence ratio of a GO MF term is defined as the number of proteins annotated this terms divided by the total number of proteins in the set.
[c] The unique ratio of a GO MF term is defined as the occurrence ratio of a GO MF term divided by the occurrence ratio in 27 species genome set.
[d] The proteins of module templates represent the core component proteins in module families with interface evolution score (*IES*) ≥ 7 and at least one GO MF term annotation in GO database.

## Supplementary Table S1. The 160 essential GO molecular function (MF) terms. (Continued)

| GO ID | GO term | Classification | Number of proteins (27 species genomes [a]) | Occurrence ratio [b] (27 species genomes; total 160,598 proteins) | Number of essential proteins | Occurrence ratio (essential proteins; total 8,364 proteins) | Unique ratio [c] (essential proteins) | Hypergeometric *P*-value (essential proteins) | Number of proteins in templates (*IES* ≥ 7) [d] | Occurrence ratio (*IES* ≥ 7; total 1,204 proteins) | Unique ratio (*IES* ≥ 7) |
|---|---|---|---|---|---|---|---|---|---|---|---|
| GO:0004716 | receptor signaling protein tyrosine kinase activity | Others | 31 | 0.0002 | 5 | 0.0006 | 3.0970 | 2E-2 | 2 | 0.00166 | 8.598474 |
| GO:0009381 | excinuclease ABC activity | Others | 50 | 0.0003 | 8 | 0.0010 | 3.0722 | 4E-3 | 0 | 0 | 0 |
| GO:0046332 | SMAD binding | Others | 45 | 0.0003 | 7 | 0.0008 | 2.9868 | 8E-3 | 6 | 0.004979 | 17.77018 |
| GO:0050136 | NADH dehydrogenase (quinone) activity | Others | 33 | 0.0002 | 5 | 0.0006 | 2.9093 | 3E-2 | 0 | 0 | 0 |
| GO:0005200 | structural constituent of cytoskeleton | Others | 210 | 0.0013 | 31 | 0.0037 | 2.8344 | 2E-7 | 11 | 0.009129 | 6.981142 |
| GO:0017056 | structural constituent of nuclear pore | Others | 37 | 0.0002 | 5 | 0.0006 | 2.5947 | 4E-2 | 1 | 0.00083 | 3.602063 |
| GO:0015087 | cobalt ion transmembrane transporter activity | Others | 45 | 0.0003 | 6 | 0.0007 | 2.5601 | 3E-2 | 0 | 0 | 0 |
| GO:0004326 | tetrahydrofolylpolyglutamate synthase activity | Others | 53 | 0.0003 | 7 | 0.0008 | 2.5360 | 2E-2 | 0 | 0 | 0 |
| GO:0008047 | enzyme activator activity | Others | 103 | 0.0006 | 13 | 0.0016 | 2.4234 | 3E-3 | 3 | 0.00249 | 3.881835 |
| GO:0004514 | nicotinate-nucleotide diphosphorylase (carboxylating) activity | Others | 66 | 0.0004 | 8 | 0.0010 | 2.3274 | 2E-2 | 0 | 0 | 0 |
| GO:0031177 | phosphopantetheine binding | Others | 111 | 0.0007 | 13 | 0.0016 | 2.2488 | 5E-3 | 0 | 0 | 0 |
| GO:0000287 | magnesium ion binding | Others | 1506 | 0.0094 | 176 | 0.0210 | 2.2440 | 4E-23 | 7 | 0.005809 | 0.619478 |
| GO:0030955 | potassium ion binding | Others | 89 | 0.0006 | 10 | 0.0012 | 2.1574 | 2E-2 | 0 | 0 | 0 |
| GO:0042802 | identical protein binding | Others | 1235 | 0.0077 | 132 | 0.0158 | 2.0523 | 1E-14 | 38 | 0.031535 | 4.100811 |
| GO:0000036 | acyl carrier activity | Others | 188 | 0.0012 | 20 | 0.0024 | 2.0427 | 2E-3 | 0 | 0 | 0 |

[a] The 27 species genome set (160,598 proteins annotated ≥ 1 GO MF terms) consists of 25 species in DEG and 2 species in module template set.
[b] The occurrence ratio of a GO MF term is defined as the number of proteins annotated this terms divided by the total number of proteins in the set.
[c] The unique ratio of a GO MF term is defined as the occurrence ratio of a GO MF term divided by the occurrence ratio in 27 species genome set.
[d] The proteins of module templates represent the core component proteins in module families with interface evolution score (*IES*) ≥ 8 and at least one GO MF term annotation in GO database.

**Supplementary Table S2.** **Expression datasets of 9 different tumor types assembled from NCBI GEO database used in our analysis.**

| Tissue | Tumor Type | Number of tumor samples | Number of normal samples | NCBI GEO accession number |
|---|---|---|---|---|
| **Adrenal** | Adrenocortical Carcinoma | 33 | 10 | GSE10927 |
| **Brain** | Astrocytoma | 26 | 23 | GSE4290 |
| **Brain** | Glioblastoma Multiforme | 77 | 23 | GSE4290 |
| **Brain** | Oligodendroglioma | 50 | 23 | GSE4290 |
| **Breast** | Ductal Carcinoma | 42 | 143 | GSE10780 |
| **Breast** | Ductal Carcinoma | 16 | 4 | GSE22544 |
| **Lymphoma** | Diffuse Large B-cell Lymphoma | 11 | 25 | GSE12453 |
| **Lymphoma** | Follicular Lymphoma | 5 | 25 | GSE12453 |
| **Lymphoma** | Hodgkin Lymphoma | 17 | 25 | GSE12453 |
| **Stomach** | Gastric Carcinoma | 38 | 31 | GSE13911 |

**Supplementary Table S3.** **Taxonomic divisions from NCBI taxonomy database.**

| Division group | Division code [a] | Division name [b] |
|---|---|---|
| MAM | PRI | Primates |
| | ROD | Rodents |
| | MAM | Mammals |
| VRT | VRT | Vertebrates |
| INV | INV | Invertebrates |
| PLN | PLN | Plants |
| BCT | BCT | Bacteria |
| ARC | ARC | Archaea |

[a,b] The division names and codes are derived from NCBI taxonomy database[22].

# References


1 Kanehisa, M. *et al.* KEGG for linking genomes to life and the environment. *Nucleic Acids Res.* **36**, D480-484 (2008).

2 Aranda, B. *et al.* The IntAct molecular interaction database in 2010. *Nucleic Acids Res.* **38**, D525-531 (2010).

3 Stark, C. *et al.* The BioGRID Interaction Database: 2011 update. *Nucleic Acids Res.* **39**, D698-704 (2011).

4 Xenarios, I. *et al.* DIP, the Database of Interacting Proteins: a research tool for studying cellular networks of protein interactions. *Nucleic Acids Res.* **30**, 303-305 (2002).

5 Mewes, H. W. *et al.* MIPS: analysis and annotation of genome information in 2007. *Nucleic Acids Res.* **36**, D196-201 (2008).

6 Ceol, A. *et al.* MINT, the molecular interaction database: 2009 update. *Nucleic Acids Res.* **38**, D532-539 (2010).

7 Chen, C. C., Lin, C. Y., Lo, Y. S. & Yang, J. M. PPISearch: a web server for searching homologous protein-protein interactions across multiple species. *Nucleic Acids Res.* **37**, W369-375 (2009).

8 Chen, Y. C., Lo, Y. S., Hsu, W. C. & Yang, J. M. 3D-partner: a web server to infer interacting partners and binding models. *Nucleic Acids Res.* **35**, W561-567 (2007).

9 Campillos, M., von Mering, C., Jensen, L. J. & Bork, P. Identification and analysis of evolutionarily cohesive functional modules in protein networks. *Genome Res.* **16**, 374-382 (2006).

10 Wu, X., Zhu, L., Guo, J., Zhang, D. Y. & Lin, K. Prediction of yeast protein-protein interaction network: insights from the Gene Ontology and annotations. *Nucleic Acids Res.* **34**, 2137-2150 (2006).

11 Ashburner, M. *et al.* Gene ontology: tool for the unification of biology. The Gene Ontology Consortium. *Nat. Genet.* **25**, 25-29 (2000).

12 Chen, Y. C., Chen, H. C. & Yang, J. M. DAPID: a 3D-domain annotated protein-protein interaction database. *Genome Inform.* **17**, 206-215 (2006).

13 Crick, F. Central dogma of molecular biology. *Nature* **227**, 561-563 (1970).

14 Neely, J. R. & Morgan, H. E. Relationship between carbohydrate and lipid metabolism and the energy balance of heart muscle. *Annu. Rev. Physiol.* **36**, 413-459 (1974).

15 Havel, P. J. Dietary fructose: implications for dysregulation of energy homeostasis and lipid/carbohydrate metabolism. *Nutr. Rev.* **63**, 133-157 (2005).

16 Castellanos, M., Wilson, D. B. & Shuler, M. L. A modular minimal cell model: purine and pyrimidine transport and metabolism. *Proc. Natl. Acad. Sci. U. S. A.* **101**, 6681-6686 (2004).

17 Smeitink, J., van den Heuvel, L. & DiMauro, S. The genetics and pathology of oxidative phosphorylation. *Nat. Rev. Genet.* **2**, 342-352 (2001).

18 Barrett, T. *et al.* NCBI GEO: archive for high-throughput functional genomic data. *Nucleic Acids Res.* **37**, D885-890 (2009).

19 Irizarry, R. A. *et al.* Exploration, normalization, and summaries of high density oligonucleotide array probe level data. *Biostatistics* **4**, 249-264 (2003).

20 Smyth, G. K. Linear models and empirical bayes methods for assessing differential expression in microarray experiments. *Stat. Appl. Genet. Mol. Biol.* **3**, Article3 (2004).

21 Benjamini, Y. & Hochberg, Y. Controlling the False Discovery Rate - a Practical and Powerful Approach to Multiple Testing. *J. Roy. Stat. Soc. B Met.* **57**, 289-300 (1995).

22 Sayers, E. W. *et al.* Database resources of the National Center for Biotechnology Information. *Nucleic Acids Res.* **39**, D38-51 (2011).
